# The decrease in ocean heat transport in response to global warming

**DOI:** 10.1038/s41558-023-01829-8

**Published:** 2023-10-12

**Authors:** Jennifer V. Mecking, Sybren S. Drijfhout

**Affiliations:** 1 National Oceanography Centre, Southampton, UK; 2 Ocean and Earth Science, National Oceanography Centre Southampton, University of Southampton, Southampton, UK; 3 Royal Netherlands Meteorological Institute, Utrecht, the Netherlands; 4 University of Utrecht, Utrecht, the Netherlands

**Keywords:** Climate and Earth system modelling, Physical oceanography, Physical oceanography

## Abstract

The ocean is taking up additional heat but how this affects ocean circulation and heat transport is unclear. Here, using coupled model intercomparison project phase 5/6 (CMIP5/6) climate projections, we show a future decrease in poleward ocean heat transport (OHT) across all Northern Hemisphere latitudes and south of 10° S. Most notably, the CMIP5/6 multimodel mean reduction in poleward OHT for the Atlantic at 26.5° N and Indo-Pacific at 20° S is 0.093–0.304 PW and 0.097–0.194 PW, respectively, dependent on scenario and CMIP phase. These changes in OHT are driven by decline in overturning circulation dampened by upper ocean warming. In the Southern Ocean, the reduction in poleward OHT at 55° S is 0.071–0.268 PW. The projected changes are stronger in CMIP6, even when corrected for its larger climate sensitivity. This is especially noticable in the Atlantic Ocean for the weaker forcing scenarios (shared socioeconomic pathway SSP 1-2.6/representative concentration pathways RCP 2.6), where the decrease is 2.5 times larger at 26.5° N due to a stronger decline in the Atlantic meridional overturning circulation.

## Abstract (web summary)

Projections of ocean heat transport show a decrease which is driven by a decline in overturning circulation. Such a decrease in ocean heat transport can dampen the global warming signal in Northwest Europe.

## Main

The oceans redistribute heat in the climate system by carrying the excess ocean heat uptake (OHU) in the tropics towards higher latitudes where the heat is released to the atmosphere^1^. In the Atlantic this is mainly accomplished by the Atlantic meridional overturning circulation (AMOC)^2^, associated with northward flowing surface and thermocline waters up to a depth of ~1,000 m. As a result, the South Atlantic Ocean is characterized by a unique equatorward flow leading to a cross-equatorial ocean heat transport (OHT) of ~0.5 PW (refs. ^3,4^). In the Indo-Pacific, OHT is more symmetric about the equator and dominated by subtropical cells (STCs)^5^. Northward OHT in the South Atlantic is counteracted by southward OHT in the southern Indo-Pacific, making global OHT poleward at almost every latitude^6^, regulating the climate by reducing equator-to-pole temperature gradients.

Under global warming, atmospheric heat transport (AHT) is expected to increase, as warmer air can hold more water vapour enhancing latent heat transport^7^. Simultaneous OHT changes have an opposing, weakening response, which is not evident from first principles^8^. When OHU is neglected, OHT and AHT changes are expected to largely cancel out^9^. This so-called Bjerknes compensation does not hold for a global warming scenario in which the oceans are taking up >90% of the excess heat associated with increased radiative forcing^10^ and the radiative heat imbalance at the top of the atmosphere is perturbed, implying that the sum of OHT and AHT changes^8^. Despite this, the opposite response of OHT and AHT to global warming appears robust in climate change projections^10^, consistent with the finding that changes in the sum of OHT and AHT are (much) smaller than in OHT and AHT separately^8^.

The question then arises as to how a decrease in OHT is accomplished in future climate change and to what extent it is uniform across all ocean basins. In general, warming leads to increased ocean stratification and vertical temperature gradients^11^ enhancing OHT. So, a decrease in OHT must be due to a weakened ocean circulation. Models agree that ocean circulation changes shape the response to greenhouse gas warming, especially in the North Atlantic^12,13^. A warmer and wetter atmosphere reduces heat loss from the ocean and enhances freshwater input at high latitudes^14^. As a result, convecting water masses become less dense, weakening the AMOC^15^. The weaker AMOC reduces OHT in the midlatitudes, leading to a region of weak ocean warming visible in models and observations^16,17^. The amount of AMOC reduction is strongly model-dependent and differs between coupled model intercomparison project phase 6 and 5 (CMIP6/5)^18^. OHT changes in the Indo-Pacific are less well documented, as most studies of the relation between OHT and global warming focus on the Atlantic, Arctic and Southern oceans^19–23^ or have a process-based focus^4,10,24^.

Here, we perform a detailed investigation of how OHT changes by the end of 2100 in future climate projections. The OHT is decomposed into Atlantic and Indo-Pacific basins as well as the Southern Ocean south of 34° S (Supplementary Fig. 1) and further into zonal mean (overturning) and azonal (gyre) components then finally into temperature, velocity and nonlinear contributions. Furthermore, the changes in OHT are compared using projections from both CMIP5 (ref. ^25^; Supplementary Table 1) and CMIP6 (ref. ^26^; Supplementary Table 2) archives.

## Results

**Fig. 1:**
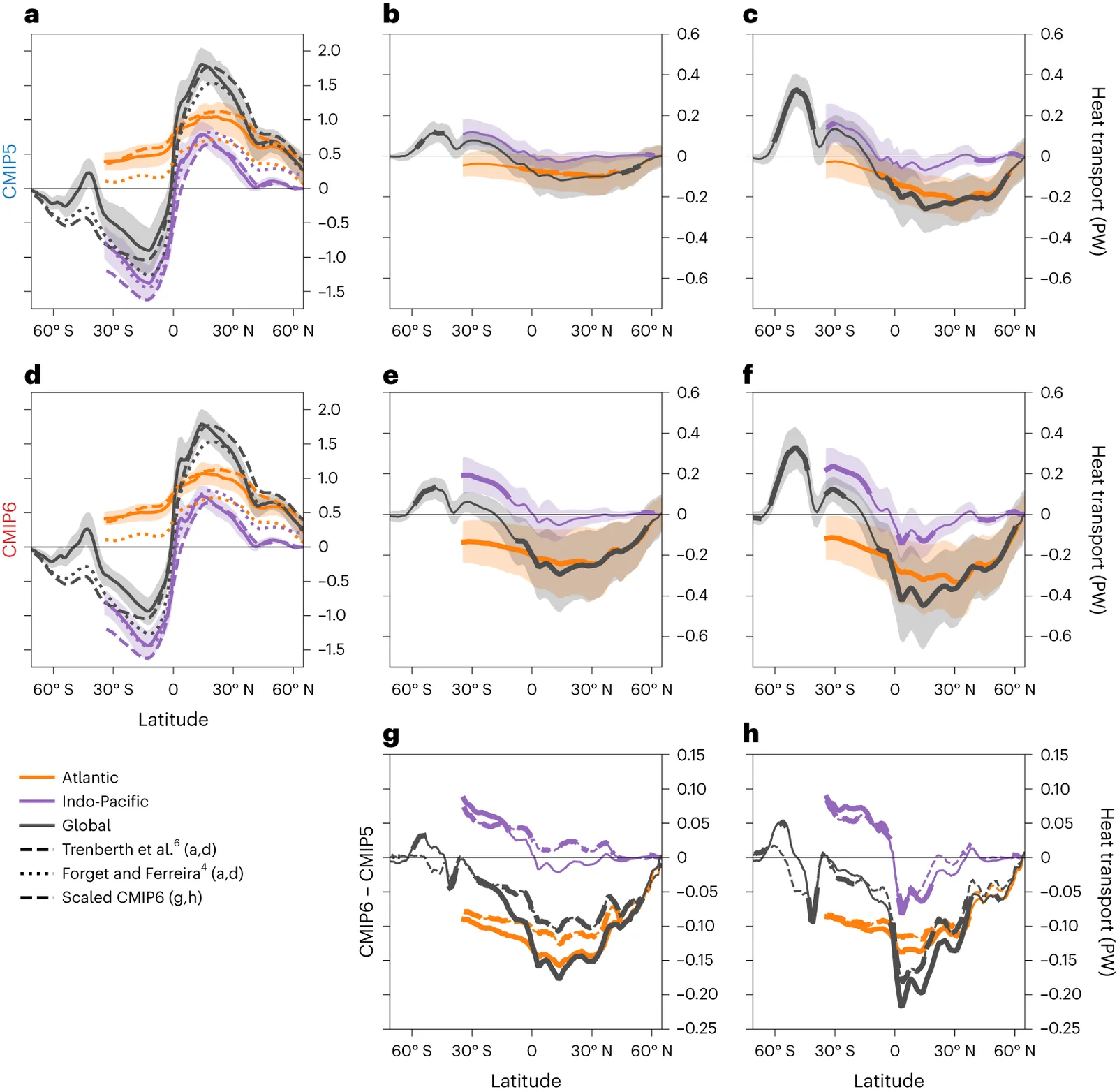
MMM of historical and changes in future climate projections of meridional OHT. **a**,**d**, MMM historical OHT for CMIP5 (**a**) and CMIP6 (**d**) models. Estimates of observation-based OHT from ref. ^6^ (dashed lines, **a**,**d**) and effective OHT estimated from reanalysis from ref. ^4^ (dotted lines, **a**,**d**). **b**,**c**,**e**,**f**, MMM of difference between RCP 2.6 and historical OHT (**b**), RCP 8.5 and historical OHT (**c**), SSP 1-2.6 and historical OHT (**e**) and SSP 5-8.5 and historical OHT (**f**). **g**, Difference between OHT changes in CMIP6 and CMIP5, including scaled CMIP6 (dashed) for RCP 2.6/SSP 1-2.6. **h**, Same as **g** but for RCP 8.5/SSP 5-8.5. The figures show OHT for global (black), Atlantic (orange) and Indo-Pacific (purple) ocean basins, with the shading indicating ±1 s.d. from the MMM (**a**–**f**). On **b**, **c**, **e**, **f**, **g** and **h**, statistical significance of the changes are indicated with the thicker lines, while a thinner line is used where the changes are not statistically significant at the 5% level.

We begin with comparing OHT in the historical reference period 1970–1999 to a reanalysis-based OHT product^4^ and observation-based estimates produced from closing the energy budget^6^ (Fig. 1a,d). The range of OHT for CMIP5 and CMIP6 falls between the observation-based and reanalysis-based estimates in the Northern Hemisphere and for the individual basins in the Southern Hemisphere, apart from south of 34° S where observations are scarce (Fig. 1a,d). It is worth noting that there are differences in the methods used to compute OHT in these estimates which could explain the discrepancies.

Because ocean circulation affects OHT it is anticipated that OHT responds differently to global warming in different ocean basins, especially in the Atlantic due to the AMOC^2^. Therefore, we divide north of 34° S OHT into contributions from the Atlantic (orange) and Indo-Pacific (purple) basins (Supplementary Fig. 1). In the Indo-Pacific basin, the multimodel mean (MMM) OHT is poleward in both hemispheres with values of 0.684/0.640 PW at 20° N and 1.215/1.244 PW at 20° S in CMIP5/6. Due to the AMOC there is northward OHT throughout the entire Atlantic basin reaching 1.001/0.998 PW at 26° N in CMIP5/6. Similar to global OHT, the Atlantic and Indo-Pacific OHT CMIP5/6 ranges fall between the observation-based and reanalysis-based estimates (Fig. 1a,d). A direct observation-based estimate of OHT at 26.5° N in the Atlantic from the RAPID mooring array indicates a northward heat transport of 1.33 PW (ref. ^27^), suggesting that the CMIP5/6 MMM (1.001/0.998 PW), as well as the reanalysis (0.72 PW) and indirect observation-based estimates (1.11 PW) all underestimate the magnitude of the Atlantic OHT.

We investigate change in OHT (2070–2099 minus 1970–1999) in the weaker RCP 2.6/SSP 1-2.6 and more extreme RCP 8.5/SSP 5-8.5 future climate scenarios of CMIP5/CMIP6. Across both scenarios the MMMs show a similar pattern of change in global OHT; a decrease in poleward OHT north of the equator and south of 10° S (Fig. 1b,c,e,f, black). The OHT in the Atlantic reduces across all latitudes by 0.093/0.232 PW in RCP 2.6/SSP 1-2.6 and 0.200/0.304 PW in RCP 8.5/SSP 5-8.5 at 26° N for CMIP5/6, while in the Indo-Pacific most changes occur in the Southern Hemisphere with a reduction in southward heat transport of 0.097/0.157 PW in RCP 2.6/SSP 1-2.6 and 0.123/0.194 PW in RCP 8.5/SSP 5-8.5 at 20° S (Fig. 1b,c,e,f, orange, purple; Table 1). The difference in response between CMIP5 and CMIP6 is striking, with CMIP6 models showing a much stronger response (Fig. 1g,h and Table 1). The largest difference occurs in Atlantic OHT in the RCP 2.6/SSP 1-2.6 scenarios where the response at 26° N in CMIP6 is 2.5 times the response in CMIP5 (Table 1). While the deduction in poleward OHT in the RCP 8.5/SSP 5-8.5 scenarios is larger, the difference between CMIP5 and CMIP6 reduces, with CMIP6 1.5 times larger (Table 1).

**Table 1:** The MMM differences in OHT at key latitudes across the ocean basins used in this study

	Atlantic 26.5° N			Indo-Pacific 20° N			Indo-Pacific 20° S			Southern Ocean 55° S		
	Total	Overturning	Gyre	Total	Overturning	Gyre	Total	Overturning	Gyre	Total	Overturning	Gyre
**Changes from historical**												
CMIP5 RCP 2.6 CMIP5 historical	**−0.093 (−9.3%)**	−0.084 (−9.6%)	−0.009 (−7.1%)	−0.019 (−2.8%)	−0.006 (−0.9%)	−0.014 (−20.7%)	0.097 (−8.0%)	0.099 (−8.7%)	−0.002 (2.6%)	**0.071 (−28.4%)**	0.049 (19.5%)	0.022 (−4.4%)
CMIP6 SSP 1-2.6 CMIP6 historical	**−0.232 (−23.3%)**	**−0.244 (−26.6%)**	0.012 (15.1%)	−0.027 (−4.2%)	−0.015 (−2.6%)	−0.011 (−17.4%)	**0.157 (−12.6%)**	**0.149 (−13.2%)**	0.008 (−6.9%)	**0.102 (−66.5%)**	**0.106 (34.5%)**	−0.003 (0.7%)
CMIP6 SSP 1-2.6 scaled CMIP6 historical	**−0.206 (−20.6%)**	**−0.222 (−24.1%)**	0.016 (20.0%)	0.004 (0.6%)	0.005 (1.0%)	−0.002 (−3.0%)	**0.142 (−11.4%)**	**0.137 (−12.1%)**	0.005 (−4.7%)	**0.072 (−46.9%)**	0.063 (20.8%)	0.009 (−1.9%)
CMIP5 RCP 8.5 CMIP5 historical	**−0.200 (−20.0%)**	**−0.185 (−21.1%)**	−0.015 (−11.6%)	−0.053 (−7.8%)	−0.028 (−4.5%)	−0.025 (−38.4%)	0.123 (−10.1%)	0.122 (−10.7%)	0.001 (−1.6%)	**0.221 (−88.3%)**	**0.224 (89.6%)**	−0.003 (0.7%)
CMIP6 SSP 5-8.5 CMIP6 historical	**−0.304 (−30.5%)**	**−0.321 (−35.0%)**	0.017 (21.2%)	−0.082 (−12.8%)	−0.051 (−9.0%)	−0.030 (−46.0%)	**0.194 (−15.6%)**	**0.179 (−15.8%)**	0.015 (−13.7%)	**0.268 (−174.5%)**	**0.313 (102.4%)**	−0.045 (9.8%)
CMIP6 SSP 5-8.5 scaled CMIP6 historical	**−0.295 (−29.5%)**	**−0.314 (−34.5%)**	0.019 (24.1%)	−0.058 (−9.0%)	−0.035 (−6.1%)	−0.023 (−34.4%)	**0.176 (−14.2%)**	**0.165 (−14.6%)**	0.011 (−10.0%)	**0.217 (−141.2%)**	**0.237 (77.7%)**	−0.021 (4.5%)
**Difference between scenarios**												
CMIP5 RCP 8.5 CMIP5 RCP 2.6	**−0.107 (115.4%)**	**−0.101 (120.7%)**	−0.006 (65.1%)	**−0.034 (174.8%)**	−0.022 (387.9%)	−0.012 (85.8%)	**0.026** (**27.0%)**	0.023 (23.3%)	0.003 (−161.3%)	**0.150 (211.5%)**	**0.175 (359.8%)**	−0.025 (115.2%)
CMIP6 SSP 5-8.5 CMIP6 SSP 1-2.6	−0.072 (31.1%)	−0.077 (31.6%)	0.005 (40.3%)	**−0.055 (207.4%)**	**−0.036 (239.6%)**	**−0.019 (164.8%)**	0.037 (23.8%)	0.030 (19.9%)	0.008 (99.9%)	**0.166 (162.2%)**	**0.207 (196.5%)**	−0.042 (1241.3%)
CMIP6 SSP 5-8.5 scaled CMIP6 SSP 1-2.6 scaled	**−0.089 (43.4%)**	−0.093 (41.7%)	0.003 (20.6%)	**−0.061 (−1734.6%)**	**−0.040 (−736.9%)**	**−0.021 (1062.6%)**	0.034 (24.3%)	0.028 (20.8%)	0.006 (114.3%)	**0.145 (201.3%)**	**0.174 (274.4%)**	−0.029 (−340.3%)
**Difference between CMIP5 and CMIP6**												
CMIP6 SSP1-2.6 − historical CMIP5 RCP2.6 − historical	**−0.139 (150.3%)**	**−0.160 (191.0%)**	0.021 (−236.6%)	−0.007 (37.7%)	−0.009 (165.8%)	0.002 (−15.9%)	**0.060 (61.4%)**	**0.050 (50.5%)**	0.010 (−496.7%)	**0.031 (44.3%)**	**0.057 (116.7%)**	−0.025 (−115.2%)
CMIP6 SSP1-2.6 Scaled − historical CMIP5 RCP2.6 − historical	**−0.113 (121.7%)**	**−0.138 (164.1%)**	**0.025 (−280.5%)**	**0.023 (−118.2%)**	0.011 (−196.2%)	0.012 (−85.7%)	**0.045 (46.0%)**	**0.038 (37.9%)**	0.007 (−369.4%)	0.001 (1.6%)	0.015 (30.2%)	−0.014 (−61.3%)
CMIP6 SSP5-8.5 − historical CMIP5 RCP8.5 − historical	**−0.105 (52.3%)**	**−0.136 (73.5%)**	**0.032 (−216.0%)**	−0.029 (54.0%)	−0.024 (85.0%)	−0.005 (19.9%)	**0.071** (**57.3%)**	**0.057** (**46.3%)**	0.014 (1194.4%)	**0.047** (**21.5%)**	**0.089** (**39.7%)**	−0.042 (1235.2%)
CMIP6 SSP5-8.5 Scaled − historical CMIP5 RCP8.5 − historical	**−0.095 (47.6%)**	**−0.129 (69.6%)**	**0.034 (−231.8%)**	−0.005 (8.5%)	−0.007 (25.6%)	0.003 (−10.2%)	**0.053 (42.9%)**	0.043 (35.2%)	0.010 (842.5%)	−0.004 (−1.7%)	0.013 (6.0%)	−0.017 (510.2%)

The leftmost column indicates what the differences are between; for each row the difference is computed as top text minus bottom text. Each entry contains two numbers, the top number is the MMM difference and the bottom number, in parentheses, indicates the percentage change (that is, top − bottom/bottom). If the text is bold the difference is significant at the 5% level.

Scaling CMIP6 data to take into account the larger climate sensitivity of CMIP6 compared to CMIP5 models (referred to as scaled CMIP6; Methods) does not have a large impact on the difference between CMIP5 and CMIP6 (Fig. 1g,h). In the SSP 1-2.6 scenario, scaling tends to reduce these differences a bit, especially in the tropical Atlantic, while in the northern Indo-Pacific they become slightly larger. In the SSP 5-8.5 scenario the impact is small, dominated by the Southern Ocean.

To further understand the details of the different responses in OHT in CMIP5/CMIP6, we decompose the changes into overturning and azonal (henceforth gyre) components and related temperature, velocity and nonlinear changes with each basin discussed separately (Methods).

### Atlantic OHT changes

**Fig. 2:**
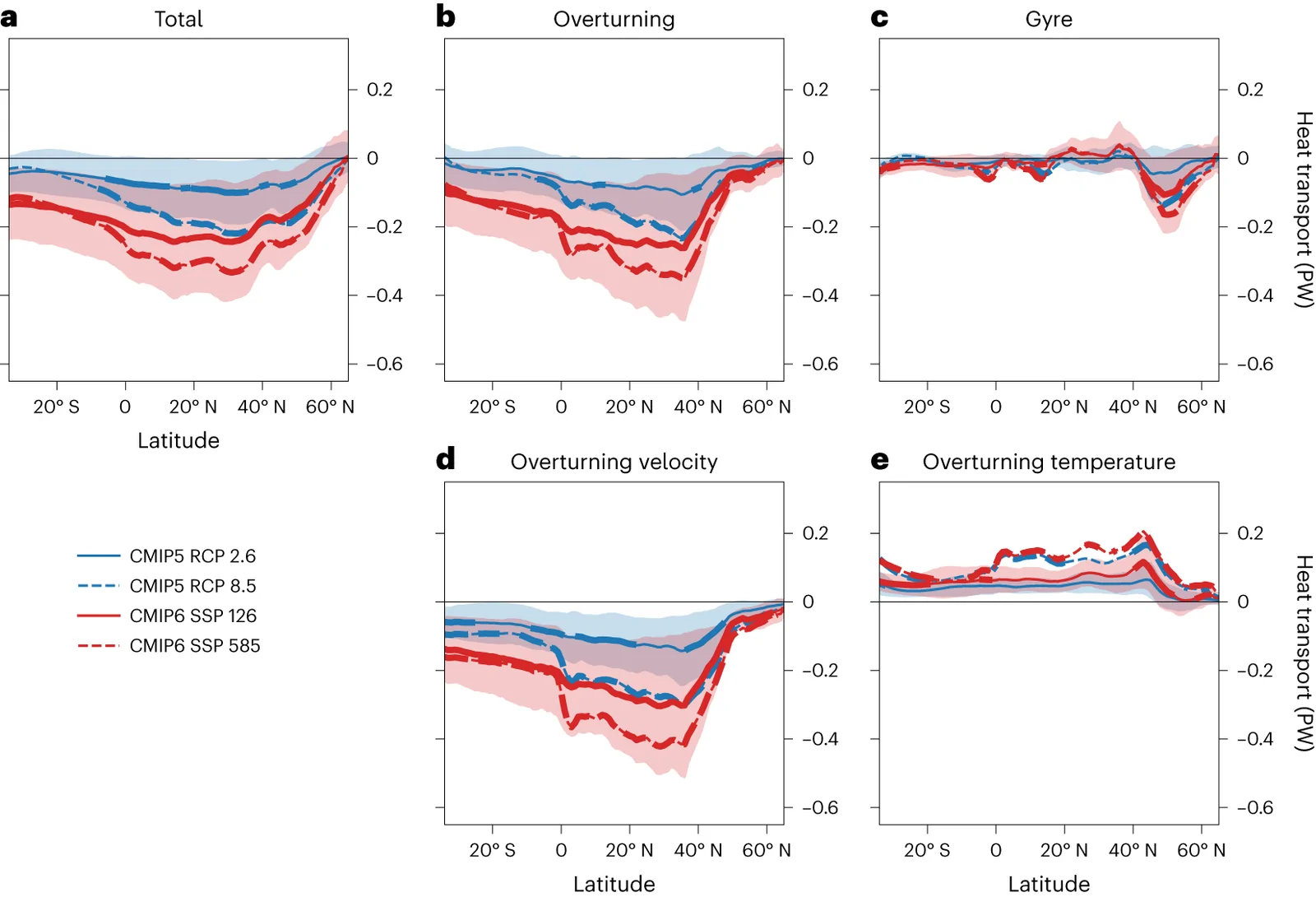
The decomposition of the changes in Atlantic meridional OHT. CMIP5 (blue) and CMIP6 (red) for the RCP 2.6/SSP 1-2.6 (solid lines) and RCP 8.5/SSP 5-8.5 (dashed lines). The shading shows ±1 s.d. for the MMM in the RCP 2.6/SSP 1-2.6 scenario. Due to the largest difference between CMIP5 and CMIP6 in RCP 2.6/SSP 1-2.6, only shading for that is shown to avoid the figure becoming too busy. **a**, Full change of the total OHT (temperature, velocity and nonlinear driven changes). **b**, Full change of the overturning OHT. **c**, Full change of the azonal OHT. **d**, Velocity-driven change of OHT. **e**, Temperature-driven overturning OHT change. Statistical significance of the changes are indicated with the thicker lines, while a thinner line is used where the changes are not statistically significant at the 5% level.

In the Atlantic, the reduction in northward OHT is mainly overturning-driven (Table 1 and Fig. 2a–c) and larger in CMIP6; at 26° N, 0.321 PW in CMIP6/SSP 5-8.5 versus 0.185 PW for CMIP5/RCP 8.5 and even more so in CMIP6/SSP 1-2.6 (0.244 PW) versus CMIP5/RCP 2.6 (0.084 PW). The gyre-driven component only plays a comparable role in the subpolar North Atlantic, with a maximum MMM reduction of <0.2 PW but the difference between CMIP5 and CMIP6 is small, 0.061 PW, in RCP 8.5/SSP 5-8.5 but larger, 0.088 PW, in RCP 2.6/SSP 1-2.6 at 50° N (Fig. 2a–c). Because the AMOC strongly projects on the gyre circulation at subpolar latitudes^28^, the gyre-driven response is consistent with the overturning-driven response here.

**Fig. 3:**
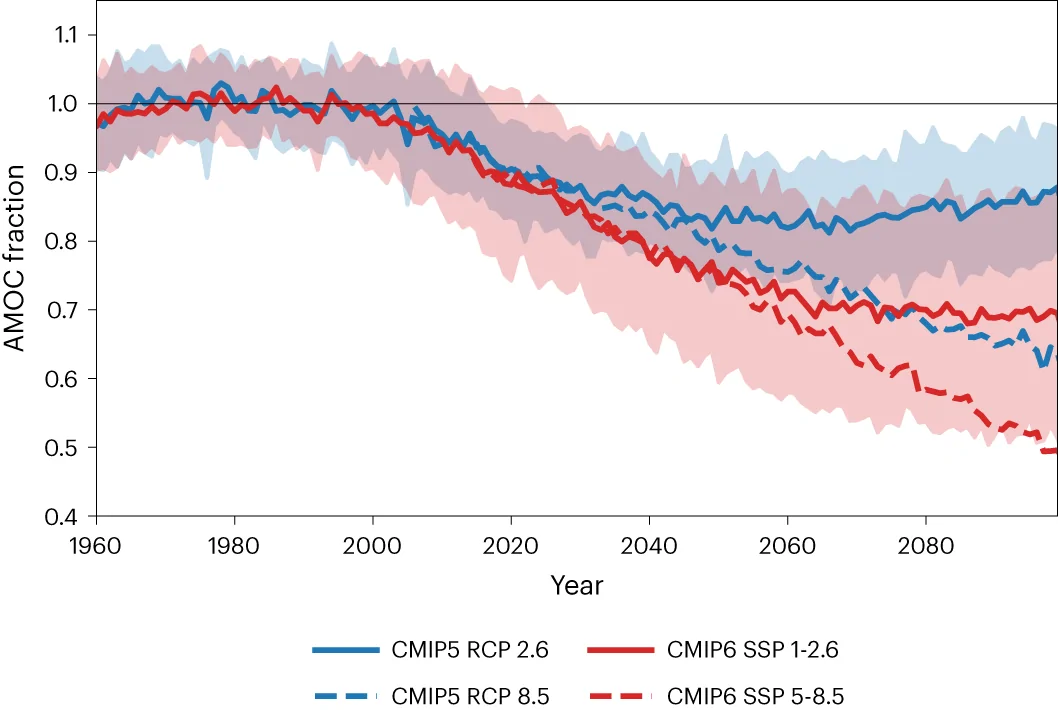
The fraction of the maximum AMOC at 26.5° N with respect to the 1970–1999 historical reference period. CMIP5 (blue) and CMIP6 (red) and future climate projections RCP 2.6/SSP 1-2.6 (solid line) and RCP 8.5/SSP 5-8.5 (dashed line). The shading shows ±1 s.d. of the RCP 2.6/SSP 1-2.6 scenarios.

**Extended Data Fig. 1:**
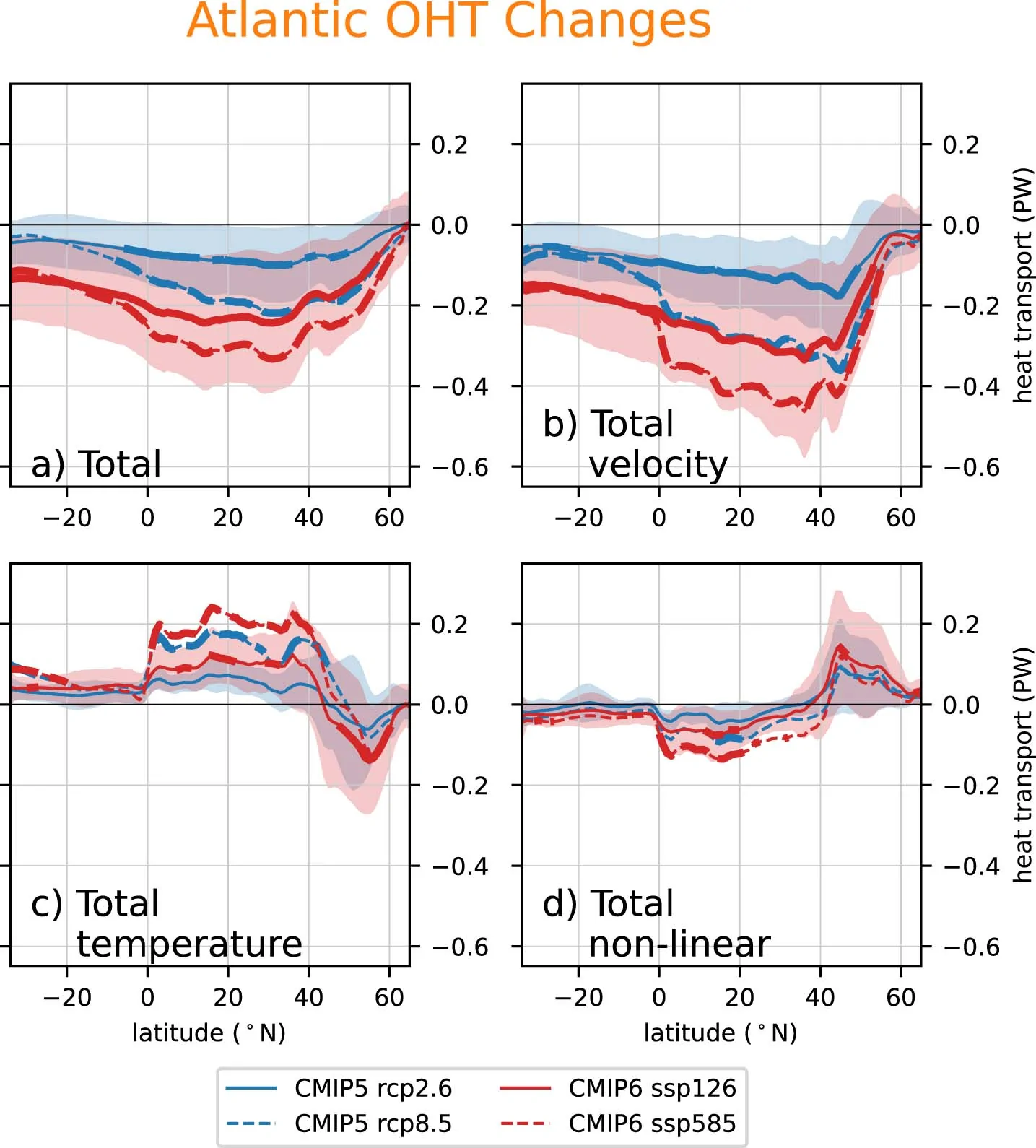
Decomposition of Total Atlantic OHT. Decomposition of changes in Atlantic OHT. **a**) Total Atlantic OHT change (overturning + gyre), **b**) the velocity-driven component, **c**) the temperature-driven component and **d**) the nonlinear component. Statistical significance of the changes are indicated with the thicker lines, while a thinner line is used where the changes are not statistically significant at the 5% level and the shading shows +/− 1 std for the MMM in the RCP2.6/SSP1-2.6 scenario.

Most of the changes in the Atlantic overturning OHT come from velocity-driven changes, which is where the largest discrepancies between CMIP5 and CMIP6 occur (Fig. 2d). The changes in velocity-driven overturning OHT are mirrored in the changes in the AMOC (Fig. 3). The temperature-driven OHT changes do not differ as much as the velocity-driven changes between the CMIP5 and CMIP6 responses (Fig. 2d,e) and dampen the velocity-driven changes south of the subpolar gyre. The total MMM nonlinear contribution in the Atlantic OHT counteracts the temperature-driven response and is less than ~0.1 PW everywhere except at the boundary between subpolar and subtropical gyre (Extended Data Fig. 1b,d).

### Indo-Pacific OHT changes

**Fig. 4:**
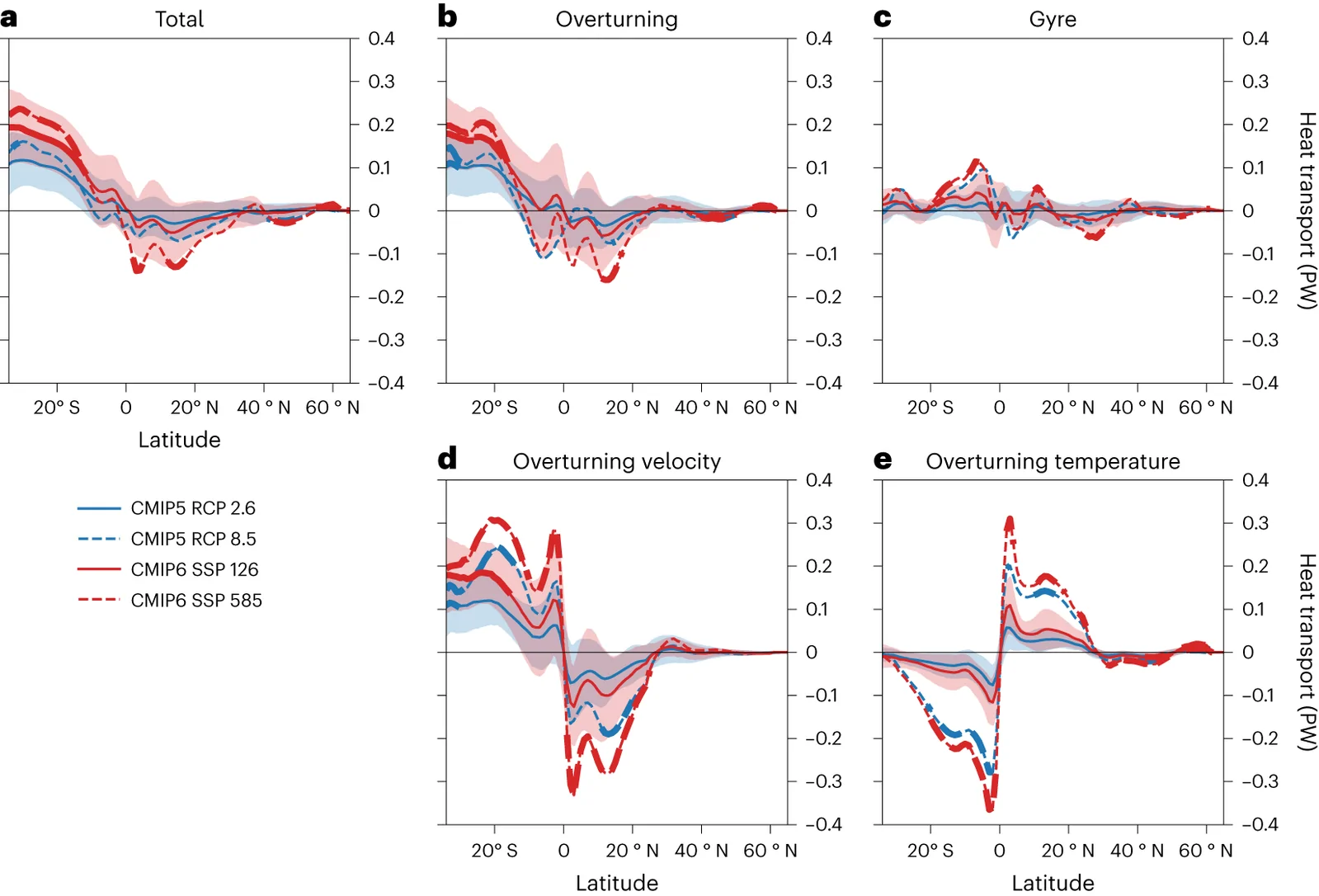
The decomposition of the changes in Indo-Pacific meridional OHT. CMIP5 (blue) and CMIP6 (red) for the RCP 2.6/SSP 1-2.6 (solid lines) and RCP 8.5/SSP 5-8.5 (dashed lines). The shading shows ±1 s.d. for the MMM in the RCP 2.6/SSP 1-2.6 scenario. Due to the largest difference between CMIP5 and CMIP6 in RCP 2.6/SSP 1-2.6 only shading for that is shown to avoid the figure becoming too busy. **a**, Full change of the total OHT (temperature, velocity and nonlinear driven changes). **b**, Full change of the overturning OHT. **c**, Full change of the azonal OHT. **d**, Velocity-driven change of OHT. **e**, Temperature-driven overturning OHT change. Statistical significance of the changes are indicated with the thicker lines, while a thinner line is used where the changes are not statistically significant at the 5% level.

**Extended Data Fig. 2:**
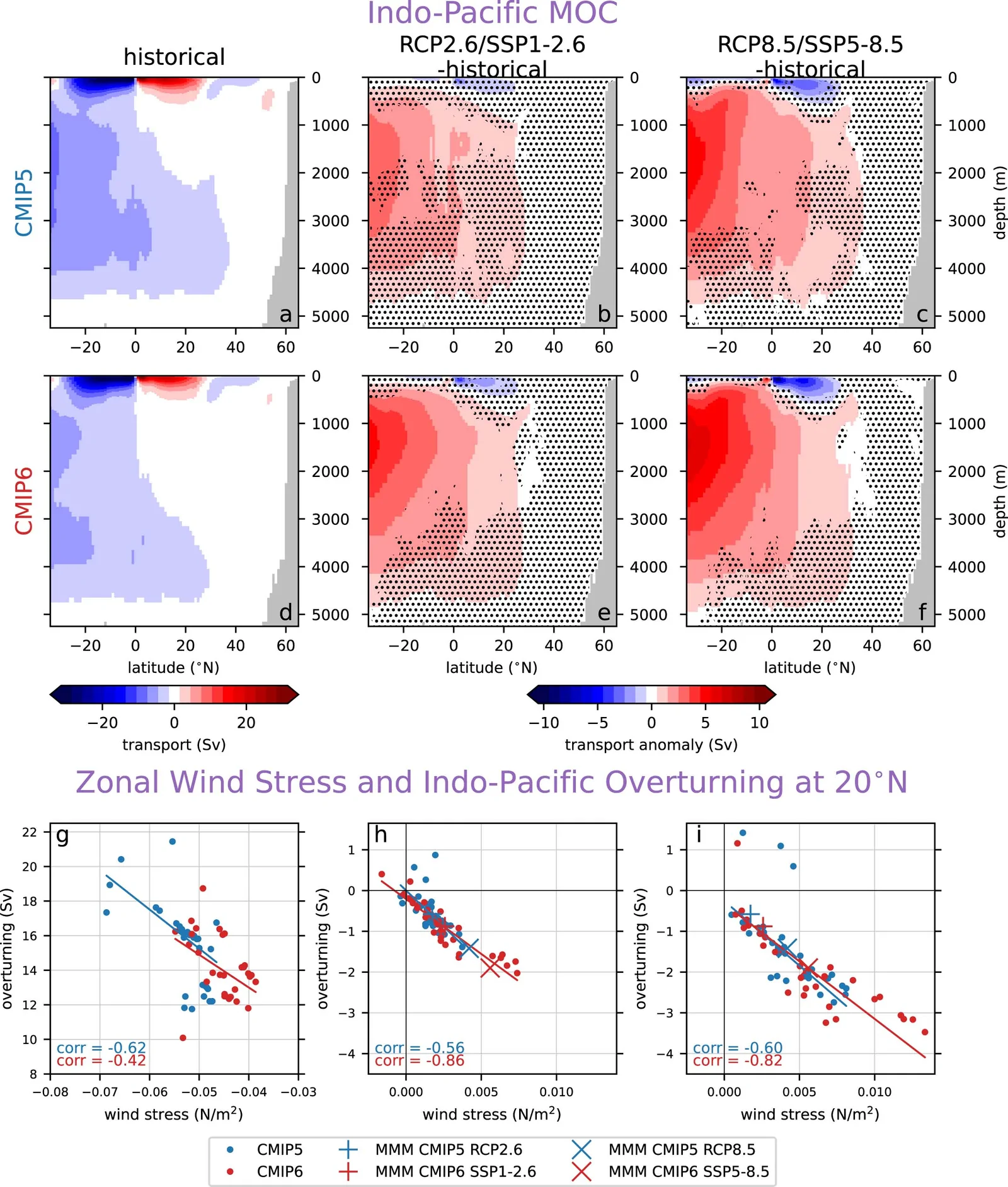
Indo-Pacific Meridional Overturning Circulation historical and future changes and relationship with wind stress. The Indo-Pacific Meridional Overturning Circulation (PMOC), historical and future projection changes. In the reference period of the historical simulation for CMIP5 (**a**) and CMIP6 (**d**) and the changes in the PMOC in future climate projections RCP2.6/SSP1-2.6 (**b**/**e**) and RCP8.5/SSP5-8.5 (**c**/**f**). Regions where the changes in PMOC are not statistically significant at the 5% level are stippled. Comparisons between the zonal mean wind stress at 20°N in the Indo-Pacific (x-axis) and the maximum PMOC at 20°N (y-axis). **g**) scatter plot between historical means, **h**) scatter plot between changes in the RCP2.6/SSP1-2.6 scenario and **i**) same as h but for RCP8.5/SSP5-8.5. The correlations are listed in the lower left corner of the figures, in blue for CMIP5 and in red for CMIP6. Note that not all models used for the OHT computations, only models with zonal wind stress on the ocean model grid are included in this figure (see Supplementary Tables 1 and 2 for details). This figure clearly shows that the decrease in (negative) overturning is due to a decrease in zonal wind stress magnitude.

**Extended Data Fig. 3:**
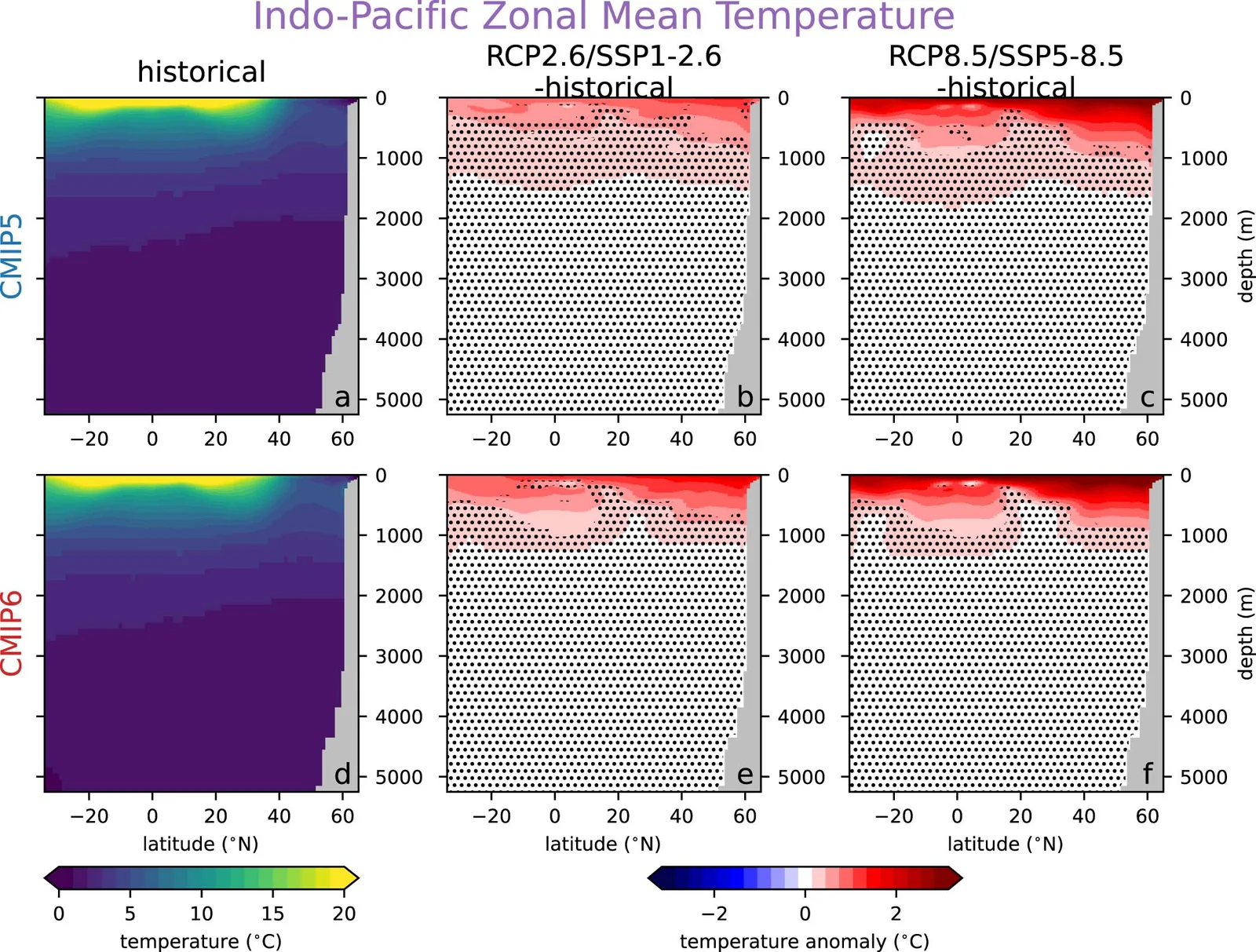
Indo-Pacific Zonal Mean Temperature. The Indo-Pacific zonal mean temperature, historical and future projection changes. In the reference period of the historical simulation for CMIP5 (**a**) and CMIP6 (**d**) and the changes in the Indo-Pacific zonal mean temperatures in future climate projections RCP2.6/SSP1-2.6 (**b**/**e**) and RCP8.5/SSP5-8.5 (**c**/**f**). Regions where the changes in zonal mean temperature are not statistically significant at the 5% level are stippled.

In the Indo-Pacific, most changes in OHT occur south of the equator with a reduction of poleward OHT of 0.097/0.157 PW for RCP 2.6/SSP 1-2.6 and 0.123/0.194 PW for RCP 8.5/SSP 5-8.5 in CMIP5/6 at 20° S (Fig. 4a). The overturning-driven component is the dominant contributor, 0.179 PW versus 0.194 PW in total at 20° S in CMIP6/SSP 5-8.5 (Table 1), while the gyre-driven component is much smaller there and only comparable in the tropics (Fig. 4b,c). In the overturning OHT change, velocity-driven changes reduce the poleward OHT in both hemispheres while temperature-driven changes counteract this reduction (Fig. 4b,d,e). In the Northern Hemisphere, the velocity-driven OHT changes are related to changes in zonal wind stress, especially in the Pacific, weakening the STC (Extended Data Fig. 2g–i). The associated decrease in OHT, however, is almost completely counteracted by the temperature-driven change that enhances OHT by the increased vertical temperature gradient resulting from global warming (Fig. 4d,e and Extended Data Fig. 3).

**Extended Data Fig. 4:**
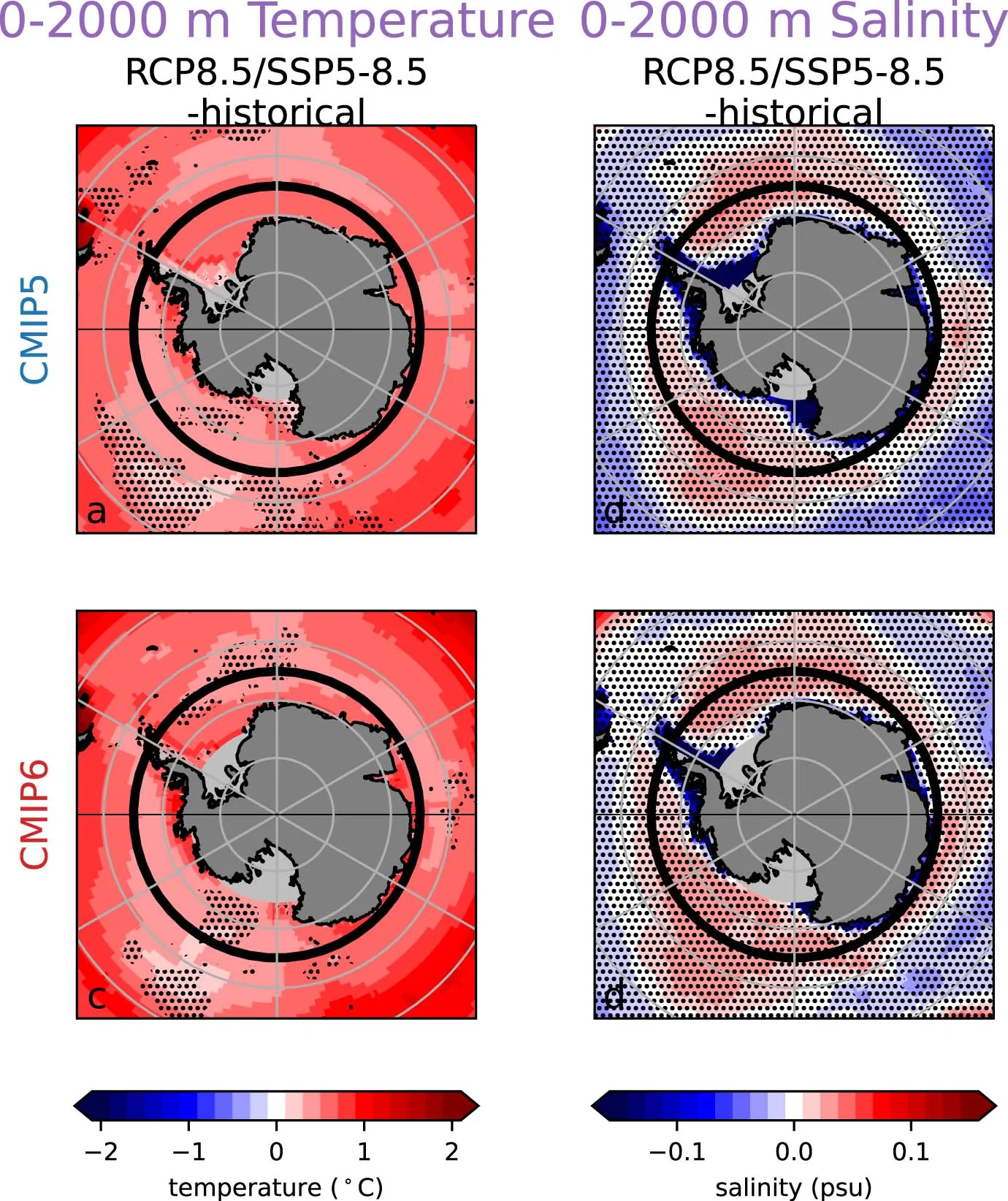
Southern Ocean Upper 2000 m Temperature and Salinity Changes. The change in Southern Ocean mean upper 2000 m temperature and salinity. Temperature (left) and salinity (right) in RCP8.5/SSP5-8.5 relative to historical for CMIP5 (top) and CMIP6 (bottom) in the reference period of the historical simulation for CMIP5 (**a**) and CMIP6 (**d**) and the changes in the temperature in future climate projections rcp2.6/ssp126 (**b**/**e**) and rcp8.5/ssp585 (**c**/**f**). Regions where the changes in temperature are not statistically significant at the 5% level are stippled. The figure shows warming and freshening of upper ocean waters on the Antarctic continental shelf, affecting AABW. Made with Natural Earth.

The situation in the Southern Hemisphere is more complex. South of ~15° S, the overturning-driven changes in OHT are dominated by the weakening of the deep overturning cell associated with less inflow of Antarctic Bottom Water (AABW)^29^ (Extended Data Fig. 2b,c,e,f). Because temperature-driven changes are mostly restricted to the upper ocean, the increasing vertical temperature gradients hardly enhance the OHT by the deep overturning cell (Extended Data Figs. 2 and 3). The weakening of the deep AABW cell is associated with reduced AABW formation^30^ associated with freshening and warming of the waters around Antarctica (Extended Data Fig. 4)^31–33^.

### Southern Ocean OHT changes

**Fig. 5:**
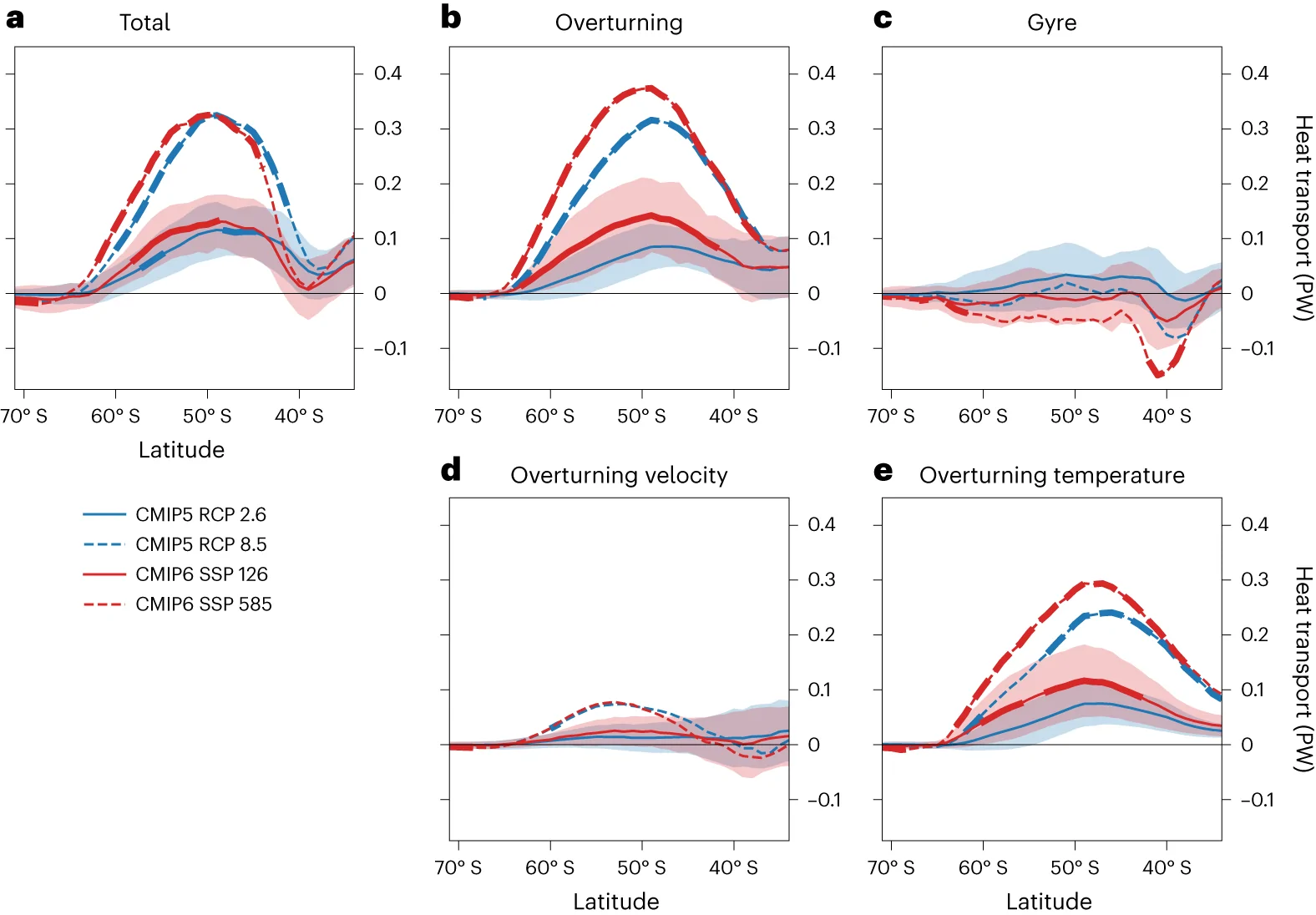
The decomposition of the changes in Southern Ocean meridional OHT. CMIP5 (blue) and CMIP6 (red) for the RCP 2.6/SSP 1-2.6 (solid lines) and RCP 8.5/SSP 5-8.5 (dashed lines). The shading shows ±1 s.d. for the MMM in the RCP 2.6/SSP 1-2.6 scenario. Due to the largest difference between CMIP5 and CMIP6 in RCP 2.6/SSP 1-2.6 only shading for that is shown to avoid the figure becoming too busy. **a**, Full change of the total OHT (temperature, velocity and nonlinear driven changes). **b**, Full change of the overturning OHT. **c**, Full change of the azonal OHT. **d**, Velocity-driven change of OHT. **e**, Temperature-driven overturning OHT change. Statistical significance of the changes are indicated with the thicker lines, while a thinner line is used where the changes are not statistically significant at the 5% level.

**Extended Data Fig. 5:**
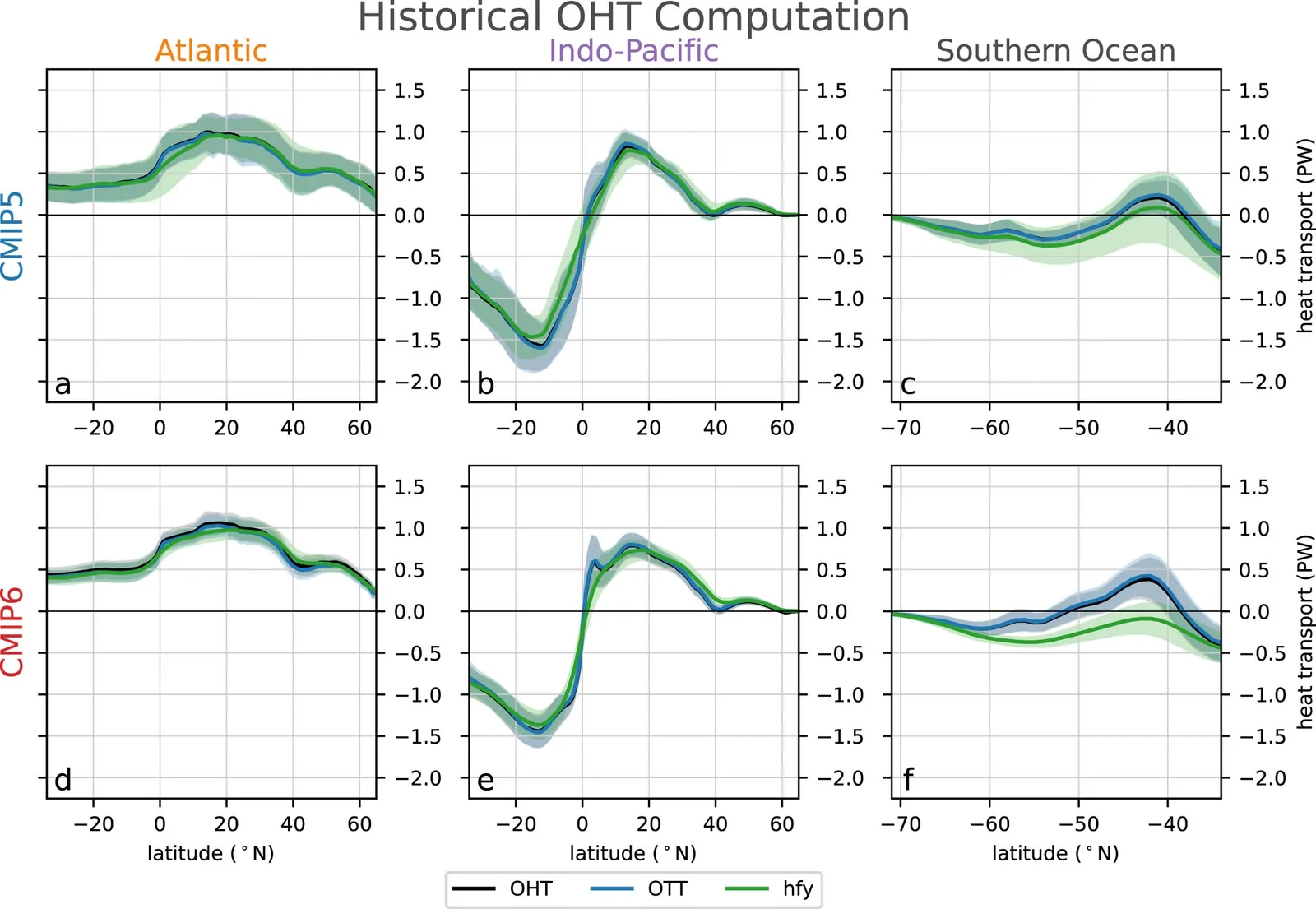
Historical OHT computation. Comparison of different was of computing OHT. OHT (black), OTT not including parameterized velocities (blue) and OTT including parameterized velocities (hfy, green). The transports for CMIP5 are in the top row (**a**–**c**) and CMIP6 in the bottom row (**d**–**f**), the left columns show transports in the Atlantic (a&d), middle in Indo-Pacific (b&e) and right column Southern Ocean (**c**&**f**). Shading indicating +/− 1 standard deviation from the MMM. Only models and ensemble members where data for all computations are available are included in this figure (see Supplementary Tables 1 and 2 for details).

**Extended Data Fig. 6:**
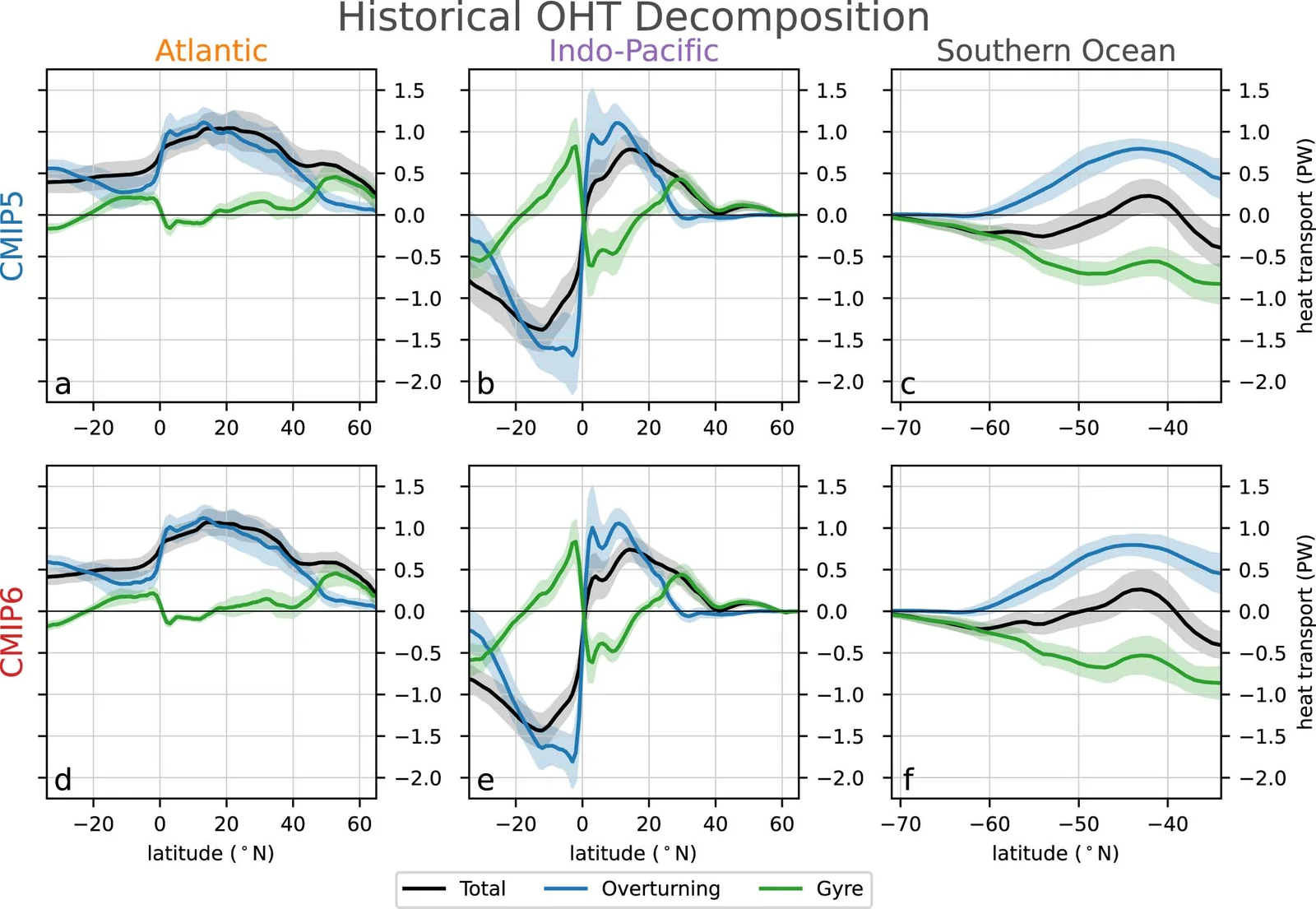
Historical OHT decomposition. The decomposition of the total historical OHT. Total (black), overturning (green) and gyre (blue) OHT, for the Atlantic (**a**,**d**), Indo-Pacific (**b**,**e**) and Southern Ocean (**c**,**f**) basins and CMIP5 (**a**,**b**,**c**) and CMIP6 (**d**,**e**,**f**). Shading indicating +/− 1 standard deviation from the MMM.

In the Southern Ocean the MMM OHT has southward transport south of 45° S and northward transport around 40° S while the observation-based and reanalysis-based OHT show southward transport throughout the Southern Ocean with stronger southward transport south of 45° S than in models (Fig. 1a,d). It should be noted that the MMM OHT does not include the bolus transport (the parameterized effects of eddies). A few models have provided ocean temperature transport (OTT) computed with bolus velocity; in these models OTT is southward over most of the Southern Ocean (Extended Data Fig. 5c,f), becoming more consistent with observations (Methods). In future climate projections the southward OHT is reduced (Fig. 1). The decrease in OHT at 55° S is large enough in the CMIP6/SSP 5-8.5 scenario to change the direction of the 0.154 PW southward to 0.114 PW northward (0.063 PW in scaled CMIP6; Table 1). While the overturning-driven component of the OHT is northward at 55° S, the gyre-driven OHT is southward and larger than the overturning-driven OHT (Extended Data Fig. 6). In future projections, the northward overturning-driven OHT at 55° S increases by 0.049/0.106 PW in the RCP 2.6/SSP 1-2.6 scenario and by 0.224/0.313 PW in the RCP 8.5/SSP 5-8.5 scenario in CMIP5/6, while the gyre-driven component shows no significant change (Fig. 5a–c and Table 1). When corrected for climate sensitivity the differences between CMIP5 and CMIP6 OHT change in the Southern Ocean become almost zero near 55° S and are no longer significant (Figs. 1g,h and 2).

**Extended Data Fig. 7:**
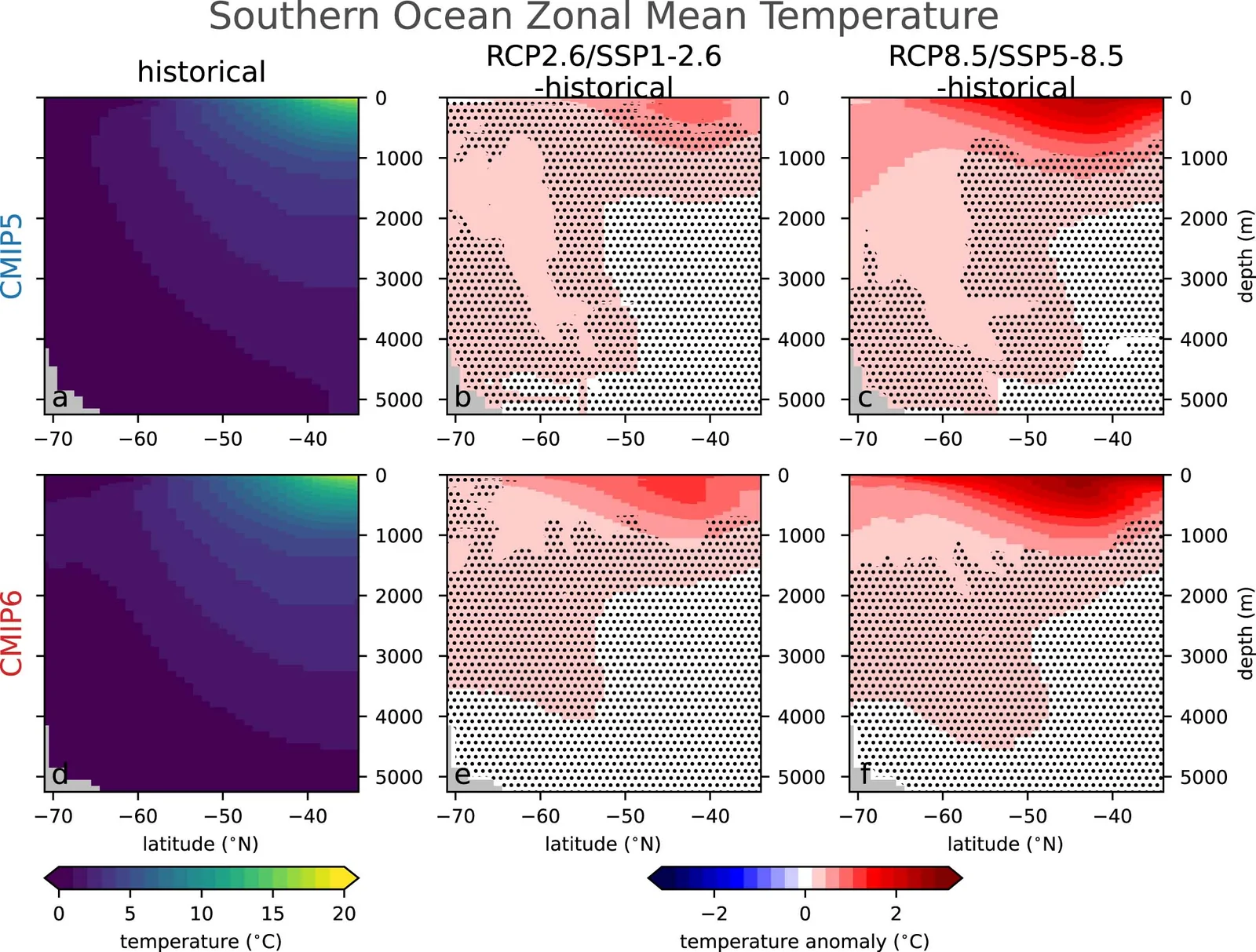
Southern Ocean Zonal Mean Temperature. The Southern Ocean zonal mean temperature, historical and future projection changes. In the reference period of the historical simulation for CMIP5 (**a**) and CMIP6 (**d**) and the changes in the Southern Ocean zonal mean temperatures in future climate projections rcp2.6/ssp126 (**b**/**e**) and rcp8.5/ssp585 (**c**/**f**). Regions where the changes in Southern Ocean zonal mean temperature are not statistically significant at the 5% level are stippled.

The dominating factor in the overturning-driven OHT change is caused by the increased vertical gradient in zonal mean temperature (Fig. 5d,e and Extended Data Fig. 7). In all scenarios, the upper layers of the Southern Ocean experience a warming peaking around 45° S (Extended Data Fig. 7). Since the overturning component of the OHT changes is largely temperature-driven, it is impacted more by the climate sensitivity, leading to CMIP5 and scaled CMIP6 OHT changes having very little difference south of ~50° S (Figs. 1g,h and 2).

## Discussion

**Extended Data Fig. 8:**
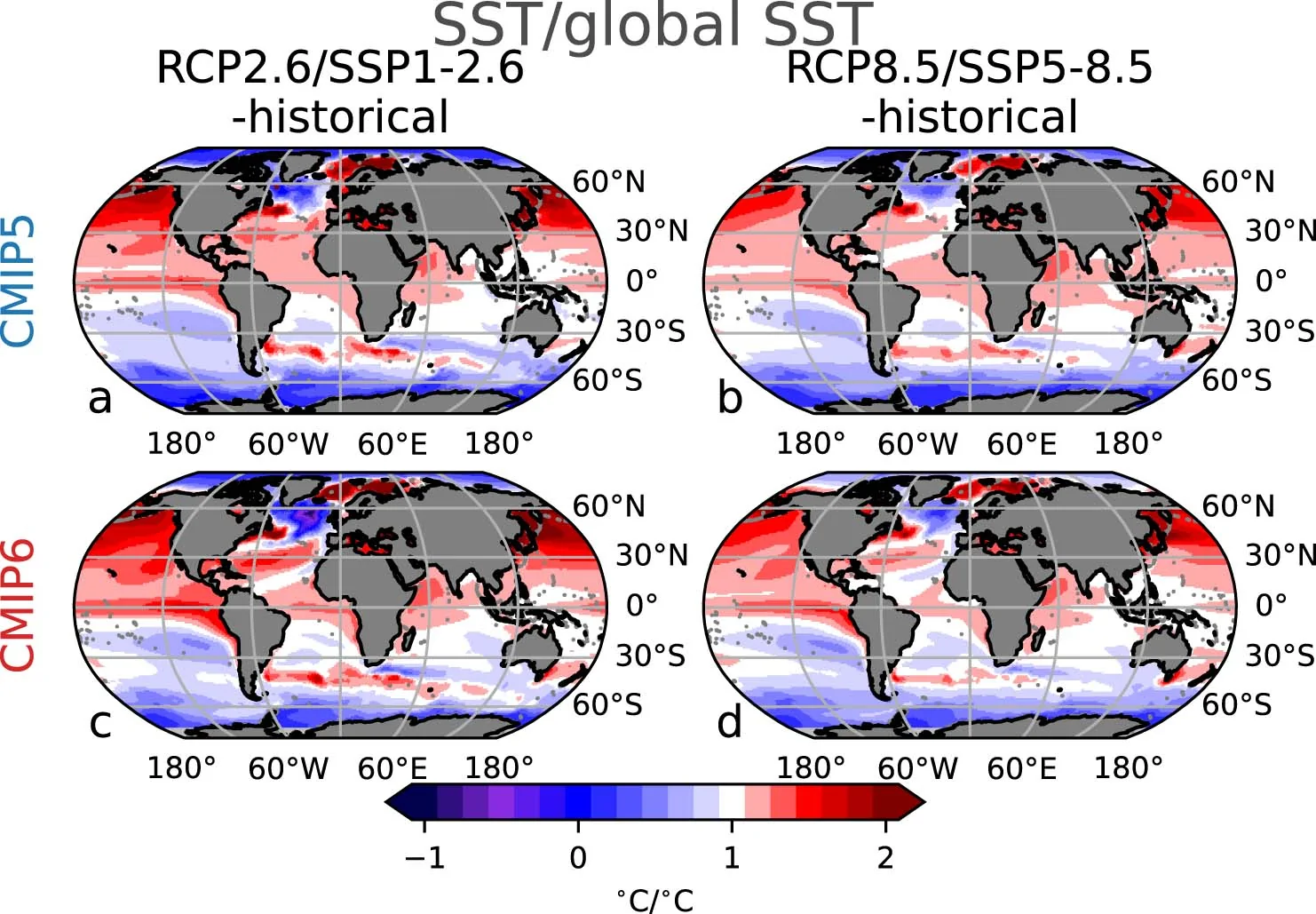
Change in SST scaled by global mean change in SST. Change in SST between future climate scenarios and historical simulation divided by the change in global mean SST. RCP2.6 in CMIP5 (**a**), SSP1-2.6 in CMIP6 (**c**), RCP8.5 in CMIP5 (**b**) and SSP5-8.5 in CMIP6 (**d**). Made with Natural Earth.

The projected reduction in poleward OHT leads to a reduction in the polar amplification of global warming which can be seen in sea surface temperatures (Extended Data Fig. 8). In the Atlantic, the reduction in OHT is relatively constant at all latitudes (including the Southern Hemisphere) leading to very little divergence in OHT until 40° N from where the changes in OHT gradually reduce to zero at 65° N (Fig. 2a). The divergence in the reduction in OHT leads to the well-known warming hole^16^. Furthermore, the reduction of temperature in the warming hole reduces the heat loss to the atmosphere in this region.

The differences between the responses in CMIP5 and CMIP6 imply that CMIP6 projects a larger response to future climate change and these differences are significant at most latitudes (Fig. 1g,h and Table 1). The largest changes are seen in the Atlantic OHT, where the differences between CMIP5 and CMIP6 are dominated by the stronger response of the AMOC to the future climate projections in CMIP6 (ref. ^18^). Whether or not this response is more realistic is still an open question but there are indications that CMIP6 models may be overly sensitive to aerosol forcing, enhancing the projected AMOC decline relative to CMIP5 (ref. ^34^). On the other hand, proxy reconstructions^35^ and a proxy-based stability analysis of the meridional overturning circulation (MOC)^36^ suggest stronger AMOC sensitivity to climate change than shown in both CMIP5 and CMIP6 historical simulations. As long as this question is not answered, the CMIP6 projections imply a strong warning that Atlantic circulation changes in response to global warming may have a stronger impact on climate than previously thought.

**Extended Data Fig. 9:**
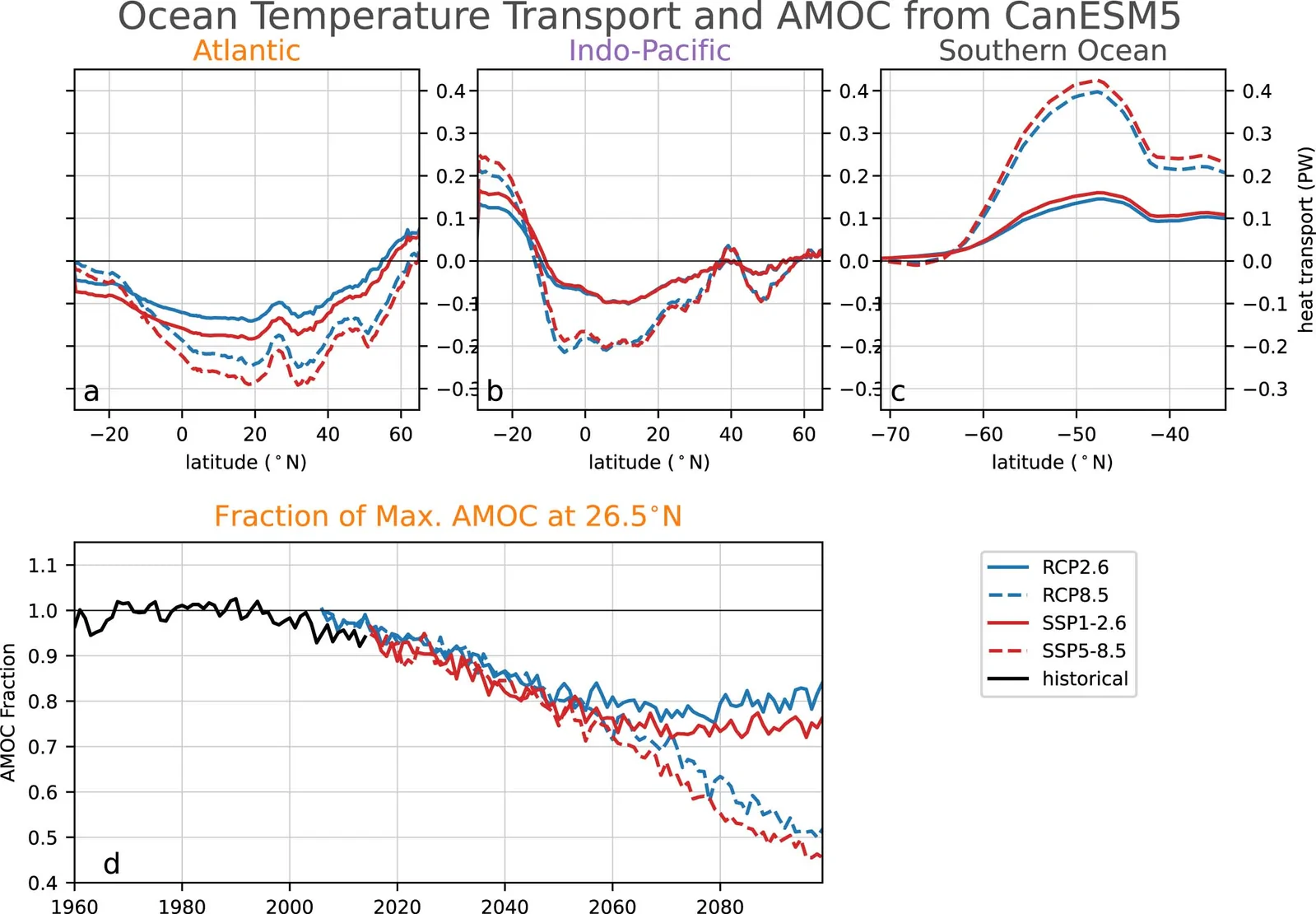
RCP and SSP comparison of CanESM5. The ensemble mean change (using 10 ensemble members) in OTT and AMOC for CanESM5. **a**) Atlantic, **b**) Indo-Pacific and **c**) Southern Ocean as well as **d**) the fraction of the maximum AMOC at 26.5°N with respect to the 1970–1999 historical reference period for the forcing from RCP2.6 (blue solid lines), RCP8.5 (blue dashed lines), SSP1-2.6 (red solid lines) and SSP5-8.5 (red dashed lines). Due to the limited data availability of the RCP2.6 and RCP8.5 scenarios the AMOC is computed from the streamfuction provided by the model (msftmz), similarly the OTT is from the OTT provided by the model (hfbasin).

An important result of our study is the larger decrease in OHT projected in CMIP6 relative to CMIP5 when comparing similar scenarios with equal amounts of radiative forcing by 2100. Some of these differences could be explained by SSP scenarios having a faster increase in radiative forcing in the early years compared to previous RCP scenarios partly due to them starting in 2015 as opposed to 2006. We found one model (CanESM5) that simulated both SSP and RCP scenarios and, indeed, changes in AMOC and OHT were larger in SSP scenarios (Extended Data Fig. 9). The difference in CanESM5 between the CMIP5 and CMIP6 scenarios, however, was much smaller than featured in the MMM ensemble. For instance, in the Atlantic at 26° N the CMIP6/SSP 5-8.5 decline in OHT is a factor of 1.5 larger than CMIP5/RCP 8.5 and a factor 2.5 larger in SSP 1-2.6/RCP 2.6. For CanESM5 these numbers are 1.2 and 1.4, respectively. We see the largest differences between CMIP5 and CMIP6 in the Atlantic basin, where the changes in OHT are driven by the AMOC. The AMOC in CanESM5 declines less in RCP scenarios (Extended Data Fig. 9d) with SSP 5-8.5 decline a factor of 1.1 larger than RCP 8.5 and 1.2 for SSP 1-2.6/RCP 2.6 in the 2070–2099 mean, while these factors are 2.0 and 1.3 for the MMM ensemble, respectively. Two recent studies have shown that in historical simulations differences in the AMOC between CMIP5 and CMIP6 as well as among CMIP6 models can be related to how models handle aerosols^34,37^. Therefore, we propose that a changed sensitivity of OHT to radiative forcing between CMIP5 and CMIP6 is the main cause for the differences in response in OHT between the two CMIP phases.

**Extended Data Fig. 10:**
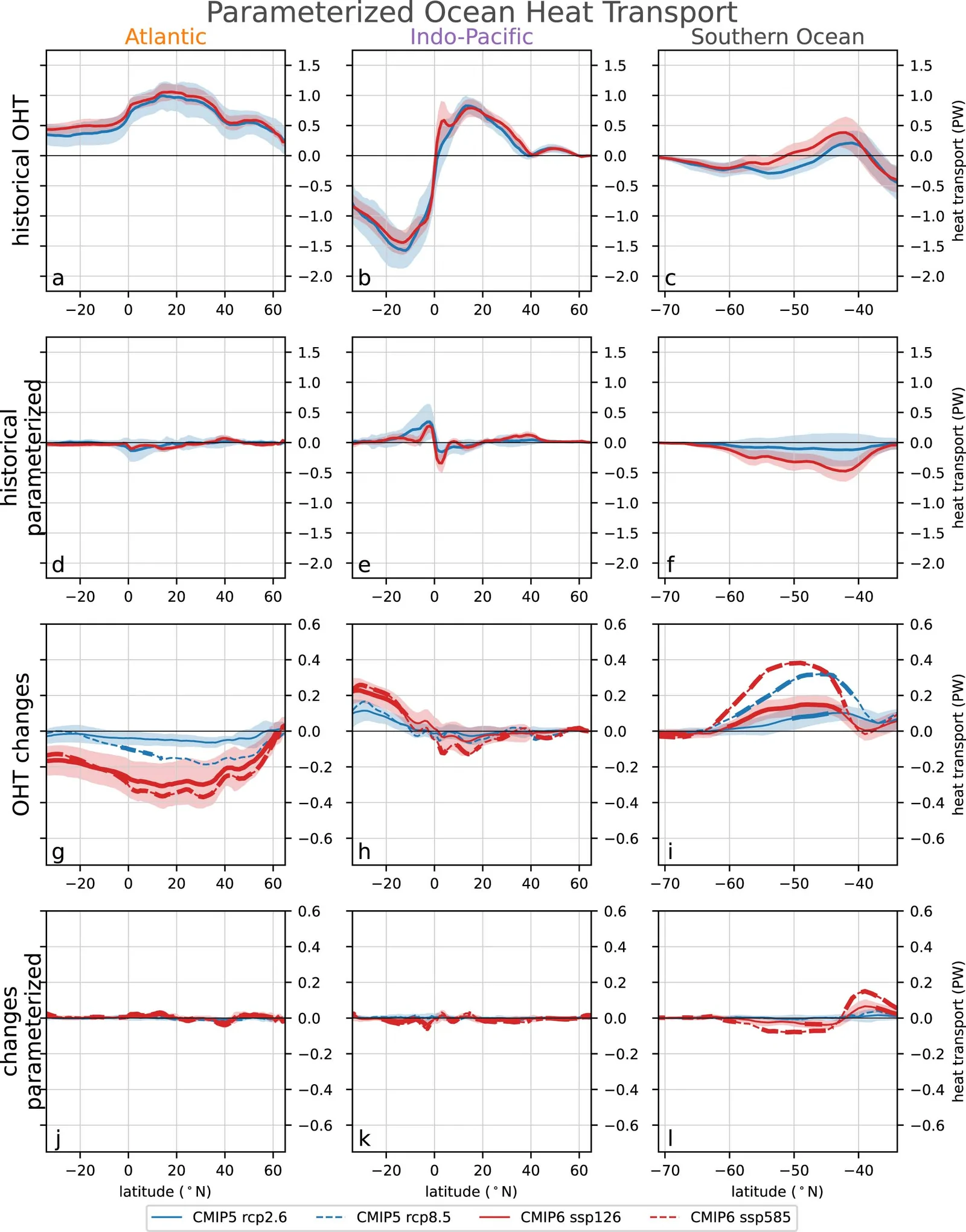
Parameterized OHT. The parameterized OTT in historical simulations and future projection changes. The historical simulation (top row) and changes in the OHT due to parameterized velocity for all three ocean basins (bottom row) for the Atlantic (left), Indo-Pacific (middle) and Southern Ocean (right) basins. CMIP5 (blue) and CMIP6 (red) for the RCP2.6/SSP1-2.6 (solid lines) and RCP8.5/SSP5-8.5 (dashed lines), the shading shows +/− 1 std for the MMM in the historical simulation (top row) and RCP2.6/SSP1-2.6 scenario (bottom row).

In the Southern Ocean, temperature-driven changes appear more robust than velocity-driven changes, as they are linked with the important role of the Southern Ocean in global OHU^38,39^. It is argued^38,39^ that the sensitivity to changing winds displayed in CMIP5/6 must be viewed with low confidence. In particular, in higher resolution models both the gyre (Antarctic Circumpolar Current) and overturning circulation show eddy-saturation^40,41^. For the Antarctic Circumpolar Current the increased amount of kinetic energy input by increasing winds is then transferred into increased eddy-kinetic energy instead of energy of the mean flow as occurs in lower-resolution models. While the wind increases the Deacon cell in the Southern Ocean, a counteracting eddy-driven overturning cell also increases in those models leaving a negligible net overturning response. These effects are largely absent in lower-resolution models in which these eddies are parameterized, resulting in relatively little change in OHT due to parameterized velocities (Extended Data Fig. 10j,l). We infer that the velocity-driven change in Southern Ocean OHT is probably overestimated in CMIP5/6 but as the temperature-driven effect is much larger, the resulting bias in projected OHT change is probably small.

## Conclusions

In the Atlantic, at all latitudes up to 65° N, there is a reduction in northward heat transport with the largest reduction near 26° N ranging from 0.093 to 0.304 PW (Table 1). The reduction in northward heat transport in the Atlantic is driven by a reduction in AMOC, partly counteracted by temperature-driven changes (Fig. 2). In the Atlantic, the difference between CMIP6 and CMIP5 projected changes is largest, associated with the larger AMOC reduction in CMIP6. Correcting for the overestimated climate sensitivity in CMIP6 does not resolve this, as such correction mainly affects the temperature-driven changes. In the Indo-Pacific, the largest reduction in poleward heat transport occurs in the Southern Hemisphere and is statistically significant only for RCP 8.5/SSP 5-8.5 scenarios (largest reduction in SSP 5-8.5 at 20° S of 0.20 PW) (Fig. 1). These changes in OHT are driven by changes in overturning circulation bringing in less deep water from the south, while the warming surface layers damp this decrease. In the Northern Hemisphere Indo-Pacific, the change in OHT by STCs due to wind changes are almost completely cancelled by the warming surface layers making the net change not statistically significant (Figs. 1 and 4). In the Southern Ocean the change in OHT is the least accurate, mainly due to eddies not being resolved (Fig. 1 and Extended Data Fig. 10). The changes in OHT are driven by changes in zonal mean temperature, enhancing northward OHT by the overturning circulation (Fig. 5).

## Methods

This study investigates changes in the meridional OHTs using 52 and 52 ensemble members from 22 and 24 CMIP5 (ref. ^25^) and CMIP6 (ref. ^26^) models, respectively (Supplementary Tables 1 and 2). Data from the historical simulations and the future climate projections RCP 2.6/SSP 1-2.6 and RCP 8.5/SSP 5-8.5 are used for this study. We use all ensemble members with data available for the OHT computations on the JASMIN CEDA CMIP5/6 archive. However, not all variables were available for some of the computations; this is indicated in Supplementary Tables 1 and 2. All means and standard deviations are calculated using weighting based on the number of ensemble members available for each model (for example, a model with three ensemble members would have a weighting of one-third for each ensemble member, while a model with only one ensemble member would have a weighting of one). Furthermore, to test whether the changes/differences are significant, a two-sample weighted Kolmogorov–Smirnov test is used with a 5% significance level^42^.

OHT is computed on ocean model grids on which the data are stored in the CMIP5/6 archives, taking into account which type of model grid the models use (B or C in Supplementary Tables 1 and 2). This causes OHT to not always follow lines of constant latitude, hence the latitudes indicated are the average latitude of the section used to compute the OHT. If the model is not on a regular latitude/longitude grid the curvature in the grid typically increases close to the north pole, therefore the northernmost latitude investigated is 65° N. In addition to computing OHT globally we divide the ocean into an Indo-Pacific and Atlantic basin extending to 34° S and the Southern Ocean, south of 34° S (Supplementary Fig. 1). The OHT is computed using monthly mean temperature (*T*(*x*, *y*, *z*, *t*)) and meridional velocity (*v*(*x*, *y*, *z*, *t*)) fields. Before computing the OHT, the section average velocity $({v}^{\ast }(y,t)\,=\,\int \int v(x,y,z,t){\mathrm{d}}x{\mathrm{d}}z)$ of each latitude section is removed in each basin (Global, Atlantic and Indo-Pacific) separately, that is $\tilde{v}=v-{v}^{\ast }$. The total OHT has units of PW and is computed as follows:

$${\mathrm{OHT}}(\,y,t)=\int \int \tilde{v}T{\mathrm{d}}x{\mathrm{d}}z\times {\mathrm{cp}}\times {\rho }_{o}/{10}^{15},$$

where cp is the specific heat capacity set to 4,000 J kg^−1^ K^−1^ and *ρ*_o_ is the mean density of sea water set to 1,026 kg m^−3^. Unless otherwise stated, we use 1970–1999 as a reference period in the historical simulations and 2070–2099 as the reference period of the future climate scenarios RCP 2.6/SSP 1-2.6 and RCP 8.5/SSP 5-8.5 (chosen as 30 year periods that are common to both CMIP5 and CMIP6 simulations). When describing the changes in OHT in the future climate projections we use the difference between the future projection reference period and historical reference period.

In global and Indo-Pacific computations of OHT there are spikes at the latitudes which are impacted by Indonesian throughflow in some models. Several ocean models which do not have high enough horizontal resolution to resolve the narrow ocean channels, like the ones present in the Indonesian throughflow, artificially narrow these channels but only on either the U- or V-grid points. The information available in the CMIP5/6 archives only contains information about the grid size on the T-grid points. For the models where these spikes occur, we removed the data from the latitudes where the spikes appear.

### Overturning versus azonal

The OHT as described in the previous section is referred to as the total OHT throughout this paper. The total OHT can be decomposed into a zonal mean (referred to as overturning) component and an azonal (often referred to as gyre) component as follows:

$${\mathrm{OH{T}_{ov}}}(\,y,t)=\int \int \langle \tilde{v}\rangle \langle T\,\rangle {\mathrm{d}}x{\mathrm{d}}z,$$

for overturning OHT and

$${\mathrm{OH{T}_{az}}}(\,y,t)=\int \tilde{v}{^\prime} T{^\prime} {\mathrm{d}}x{\mathrm{d}}z,$$

for azonal OHT, where $\langle \cdot \rangle =\frac{\int \cdot {\mathrm{d}}x}{\int {\mathrm{d}}x}$ is the zonal mean and ⋅′ = ⋅ − 〈⋅〉 are the deviations from the zonal mean. The decomposition of the historical OHT into the overturning and gyre components can be seen in Extended Data Fig. 6.

### Temperature, velocity and nonlinear decomposition

The changes in OHT computed for the future climate projections for total, overturning and azonal OHT can be broken down even further into changes driven by velocity changes, temperature changes and nonlinear contributions. The velocity-driven contributions are computed by setting the temperature in the OHT calculations to the mean seasonal cycle from the historical reference period while allowing the velocity to change. Similarly, the temperature-driven changes are computed by setting the velocity to the mean seasonal cycle from the historical reference period while allowing the temperature to change. Finally, the nonlinear changes are computed as a residual by removing the velocity-based changes and temperature-based changes from the total change. Note that we have defined the 30 year reference period to have a seasonal cycle, which slightly reduces the nonlinear component of the OHT compared to using a 30 year mean. A limitation of using monthly means is that some of the smaller timescale changes in OHT are missed, in particular changes in OHT due to eddies, which are largest at the gyre boundaries and in the Southern Ocean. It has been shown for freshwater transport in higher resolution ocean models that the timescale of the data used has an impact^43,44^; however, with a few exceptions (HadGEM3-GC31-MM), most models have an ocean resolution of 1° or less. Furthermore, smaller scale circulation is often parameterized, especially in 1° ocean models. Unfortunately, for most models this parameterized velocity is not included in the ocean velocity data provided in CMIP archives. About half of the models have the meridional temperature transport computed at each grid point (referred to as hfy), which includes the parameterized transports and total OTT can be computed from that. Note that for OTT the section-averaged velocity is not removed. Comparisons of OHT and OTT computed from three-dimensional model temperatures and velocities and OTT computed from hfy show that the largest differences occur in regions where there are a lot of eddies, most notably in the Southern Ocean but also at gyre boundaries (Extended Data Fig. 5). Changes in parameterized OTT for the most part are less than 10% of total change in OHT with the exception of the Southern Ocean (Extended Data Fig. 10j,k,l).

### Meridional overturning circulation

The MOC used in this study is computed at each latitude and for each basin using the meridional velocity with the section-averaged velocity removed to isolate the overturning component of the circulation, similar to what has been done in ref. ^45^ in the Atlantic,

$${\mathrm{MOC}}(\,y,z,t)=-{\int }_{\!\!\!-H}^{z}{\int }_{\!\!\!E}^{W}\tilde{v}(x,y,z,t){\mathrm{d}}x{\mathrm{d}}z,$$

where *H* = *H* (*x*, *z*) is the ocean depth. It should be noted that most of the CMIP5/6 models have the MOC as part of the data available in the CMIP5/6 archives and that computing the overturning based on the meridional velocity (at least for the Atlantic^34^) leads to small differences in the overturning of ~1 Sv, if computed correctly. Furthermore, this study also removes the section-based velocity to have zero net transport, which will cause slight differences in the streamfunction (mainly the surface of the streamfunction being the volume transport of the section (~1 Sv) and not 0).

### CMIP6 and climate sensitivity

It is well known that the CMIP6 MMM has a higher climate sensitivity than the CMIP5 MMM^46,47^. To test whether the differences between CMIP5 and CMIP6 in OHT responses are linked to the larger climate sensitivity in CMIP6, we have scaled the CMIP6 OHT changes using global mean temperature (GMT) changes. In this way, we isolate the effect of higher temperature projections in CMIP6 and by removing it, we effectively determine the OHT changes if the GMT projections in CMIP6 would have been equal to those in CMIP5. First, the relationship between GMT changes and OHT changes in CMIP6 are computed using linear regression to obtain the value of how much OHT changes per degree of GMT change, *a*. Using the difference between GMT changes in CMIP6 and CMIP5, the MMM OHT in CMIP6 is then adjusted as follows (referred to as scaled CMIP6):

$${{\rm{OHT}}}^{{\rm{scaled}}-{\rm{CMIP}}6}={{\rm{OHT}}}^{{\rm{CMIP}}6}-a\Big({{\rm{GMT}}}^{{\rm{CMIP}}6}-{{\rm{GMT}}}^{{\rm{CMIP}}5}\,\Big)$$

Furthermore, when investigating the significance of differences between scaled CMIP6 models and CMIP5 models, the differences are scaled using the scaling factor OHT^scaled − CMIP6^/OHT^CMIP6^.

## Online content

Any methods, additional references, Nature Portfolio reporting summaries, source data, extended data, supplementary information, acknowledgements, peer review information; details of author contributions and competing interests; and statements of data and code availability are available at https://doi.org/10.1038/s41558-023-01829-8.

## Supplementary information

## Data Availability

The CMIP5 and CMIP6 data used in this study are freely available online at https://esgf-node.llnl.gov/search/cmip5/ and https://esgf-node.llnl.gov/search/cmip6/, respectively. The reanalysis-based^4^ and observation-based^6^ OHT are available on the journal article webpages at https://www.nature.com/articles/s41561-019-0333-7#data-availability and https://journals.ametsoc.org/view/journals/clim/32/14/jcli-d-18-0872.1.xml?tab_body=supplementary-materials, respectively.

## Code availability

The code used for the current study are available in the GitHub Repository^48^ at https://doi.org/10.5281/zenodo.8312934.
