# Supplementary material for: The decrease in ocean heat transport in response to global warming
Source: Nat Clim Chang. 2023 Oct 12;13(11):1229–36. doi: 10.1038/s41558-023-01829-8 (PMC13538074; doi:10.1038/s41558-023-01829-8)
Supplement: Supplementary file 1 [file 41558_2023_1829_MOESM1_ESM.pdf]

nature climate change

Article

https://doi.org/10.1038/s41558-023-01829-8

The decrease in ocean heat transport in response to global warming

In the format provided by the authors and unedited

Supplementary Information

Figure S1 2

Table S1 3

Table S2 4

References 6

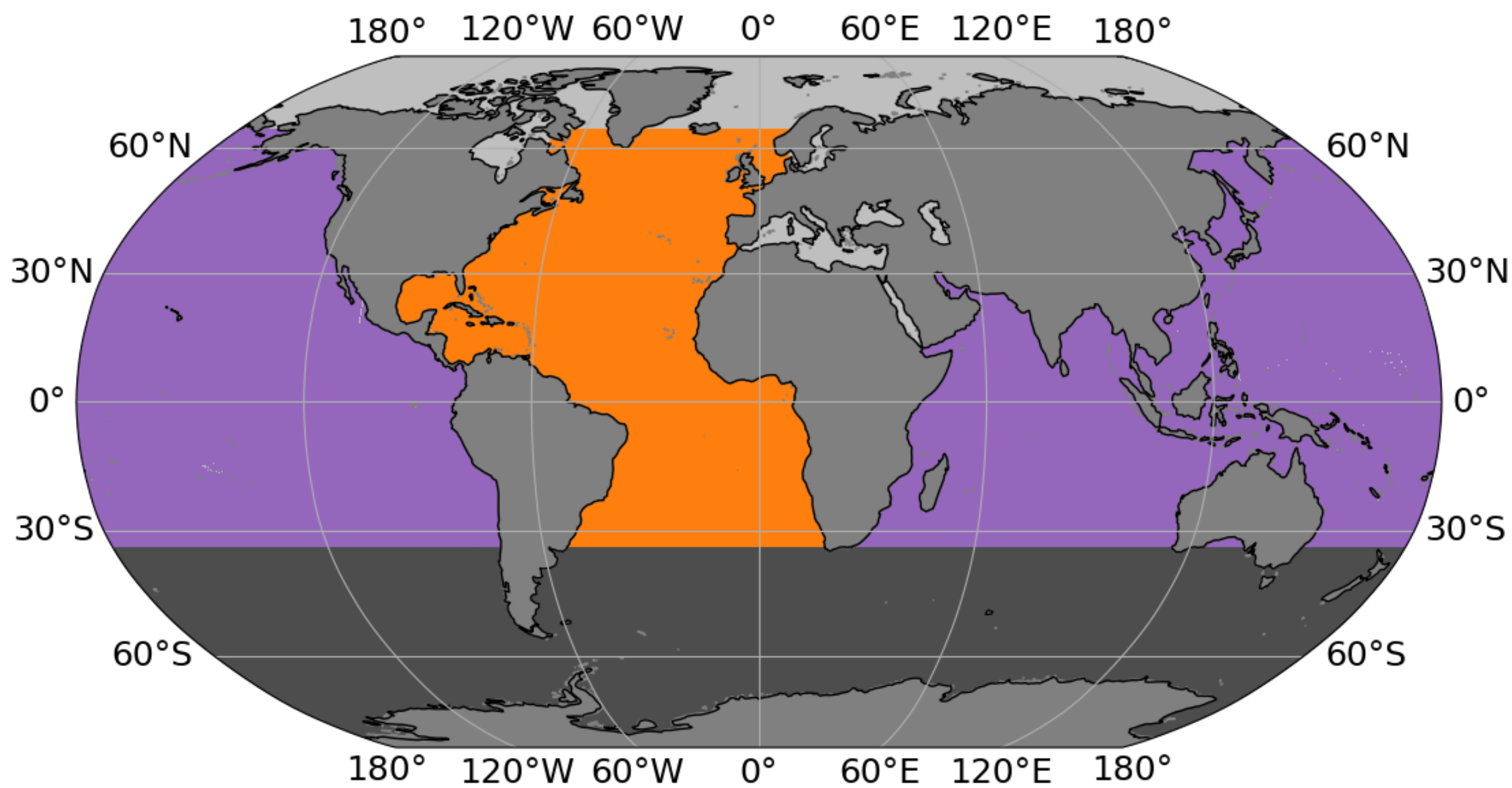

Figure S1

Ocean basins used for computations of OHT. Atlantic (orange), Indo-Pacific (purple) and Southern Ocean (dark grey). Made with Natural Earth.

Model	Institute	Ensemble Members	Ocean Model Grid
bcc-csm1-1	BCC	r1i1p1^*	B
bcc-csm1-1-m	BCC	r1i1p1^*	B
BNU-ESM	BNU	r1i1p1^*	B
CCSM4	NCAR	r1i1p1^*, r3i1p1*, r5i1p1*, r6i1p1*	B
CESM1-CAM5	NSF-DOE-NCAR	r1i1p1*, r2i1p1*, r3i1p1*	B
CNRM-CM5	CNRM-CERFACS	r1i1p1^*	C
CSIRO-Mk3-6-0	CSIRO-QCCCE	r1i1p1*, r2i1p1*, r3i1p1*, r4i1p1*, r5i1p1*, r6i1p1*, r7i1p1*, r8i1p1*, r9i1p1*, r10i1p1*	B
CanESM2	CCCma	r1i1p1*, r2i1p1*, r3i1p1*, r4i1p1*, r5i1p1*	B
EC-EARTH	ICHEC	r8i1p1*, r12i1p1*	C
FGOALS-g2	LASG-CESS	r1i1p1^*	A
GFDL-ESM2M	NOAA-GFDL	r1i1p1	B
GISS-E2-R	NASA-GISS	r1i1p1, r1i1p2, r1i1p3	C
HadGEM2-AO	NIMR-KMA	r1i1p1^*	B
HadGEM2-ES	MOHC	r2i1p1^*, r3i1p1^*, r4i1p1^*	B
IPSL-CM5A-LR	IPSL	r1i1p1, r2i1p1^, r3i1p1^, r4i1p1^	C
IPSL-CM5A-MR	IPSL	r1i1p1	C
MIROC5	MIROC	r1i1p1*, r2i1p1*, r3i1p1*	B
MPI-ESM-LR	MPI-M	r1i1p1, r2i1p1, r3i1p1	C
MPI-ESM-MR	MPI-M	r1i1p1	C
MRI-CGCM3	MRI	r1i1p1	B
NorESM1-M	NCC	r1i1p1	C
Nor-ESM1-ME	NCC	r1i1p1	C

Table S1

CMIP5 models used in this study. ^ indicates that zonal wind stress data on ocean grid are not available/has issues and * indicates that temperature data (hfy) on ocean grid are not available/has issues.

Model	Institute	Ensemble Members	Ocean Model Grid	References
BCC-CSM2-MR	BCC	r1i1p1f1^*	B	51,52,53
CAMS-CSM1-0	CAMS	r1i1p1f1*	B	54,55,56
CAS-ESM2-0	CAS	r1i1p1f1*	B	57
CESM2-WACCM	NCAR	r1i1p1f1*	B	58,59,60
CIESM	THU	r1i1p1f1*	B	61,62,63
CMCC-ESM2	CMCC	r1i1p1f1*	C	64,65,66
CNRM-CM6-1	CNRM-CERFACS	r1i1p1f2, r2i1p1f2, r3i1p1f2, r4i1p1f2, r5i1p1f2, r6i1p1f2	C	67,68,69
CNRM-ESM2-1	CNRM-CERFACS	r5i1p1f2	C	70,71,72
CanESM5	CCCma	r1i1p1f1*, r1i1p2f1^*, r2i1p1f1^*, r2i1p2f1^*, r3i1p1f1^*, r3i1p2f1^*, r4i1p1f1^*, r4i1p2f1^*, r5i1p1f1^*, r5i1p2f1^*, r6i1p2f1^*, r7i1p2f1^*, r8i1p1f1^*, r8i1p2f1^*, r9i1p2f1^*, r10i1p2f1^*	C	73,74,75,121,122
EC-Earth3	EC-Earth-Consortium	r1i1p1f1	C	76,77,78
FGOALS-f3-L	CAS	r1i1p1f1^*	B	79,80,81
FGOALS-g3	CAS	r1i1p1f1^*	B	82,83,84
FIO-ESM-2-0	FIO-QLNM	r1i1p1f1*	B	85,86,87
HadGEM3-GC31-LL	MOHC	r1i1p1f3	C	88,89,90
HadGEM3-GC31-MM	MOHC	r1i1p1f3	C	91,92,93
IPSL-CM6A-LR	IPSL	r1i1p1f1, r2i1p1f1, r3i1p1f1	C	94,95,96
MIROC6	MIROC	r1i1p1f1*, r2i1p1f1^*, r3i1p1f1^*	B	97,98,99
MPI-ESM1-2-HR	MPI-M (historical), DKRZ (ScenarioMIP)	r1i1p1f1	C	100,101,102
MPI-ESM1-2-LR	MPI-M	r1i1p1f1	C	103,104,105
MRI-ESM2-0	MRI	r1i1p1f1	B	106,107,108
NorESM2-LM	NCC	r1i1p1f1	C	109,110,111
NorESM2-MM	NCC	r1i1p1f1	C	112,113,114
TaiESM1	AS-RCEC	r1i1p1f1^*	B	115,116,117
UKESM1-0-LL	MOHC	r1i1p1f2, r2i1p1f2, r3i1p1f2, r4i1p1f2, r8i1p1f2	C	118,119,120

Table S2

CMIP6 models used in this study. ^ indicates that zonal wind stress data on ocean grid are not available/has issues and * indicates that temperature data (hfy) on ocean grid are not available/has issues. The references in the reference column are for the historical, SSP1-2.6 and SSP5-8.5 respectively. Note that there were no data references available for CAS-ESM2-0 for SSP1-2.6 and SSP5-8.5. Also, the additional 2 references for CanESM5 are for the rcp26-cmip5 and rcp85-cmip5 experiments and + indicates the ensemble members used.

References

51. Wu, T. et al. BCC BCC-CSM2MR model output prepared for CMIP6 CMIP historical. Earth System Grid Federation. Version 20181126. (2018). doi:https://doi.org/10.22033/ESGF/CMIP6.2948
52. Xin, X. et al. BCC BCC-CSM2MR model output prepared for CMIP6 ScenarioMIP ssp126. Earth System Grid Federation. Version 20190319. (2019). doi:https://doi.org/10.22033/ESGF/CMIP6.3028
53. Xin, X. et al. BCC BCC-CSM2MR model output prepared for CMIP6 ScenarioMIP ssp585. Earth System Grid Federation. Version 20190319. (2019). doi:https://doi.org/10.22033/ESGF/CMIP6.3050
54. Rong, X. CAMS CAMS_CSM1.0 model output prepared for CMIP6 CMIP historical. Earth System Grid Federation. Version 20190708. (2019). doi:https://doi.org/10.22033/ESGF/CMIP6.9754
55. Rong, X. CAMS CAMS-CSM1.0 model output prepared for CMIP6 ScenarioMIP ssp126. Earth System Grid Federation. Version 20190708. (2019). doi:https://doi.org/10.22033/ESGF/CMIP6.11046
56. Rong, X. CAMS CAMS-CSM1.0 model output prepared for CMIP6 ScenarioMIP ssp585. Earth System Grid Federation. Version 20190708. (2019). doi:https://doi.org/10.22033/ESGF/CMIP6.11052
57. Chai, Z. CAS CAS-ESM1.0 model output prepared for CMIP6 CMIP historical. Earth System Grid Federation. Version 20201228. (2020). doi:https://doi.org/10.22033/ESGF/CMIP6.3353
58. Danabasoglu, G. NCAR CESM2-WACCM model output prepared for CMIP6 CMIP historical. Earth System Grid Federation. Version 20190917. (2019). doi:https://doi.org/10.22033/ESGF/CMIP6.10071

59. Danabasoglu, G. NCAR CESM2-WACCM model output prepared for CMIP6 ScenarioMIP ssp126. Earth System Grid Federation. Version 20210211. (2019). doi:https://doi.org/10.22033/ESGF/CMIP6.10100
60. Danabasoglu, G. NCAR CESM2-WACCM model output prepared for CMIP6 ScenarioMIP ssp585. Earth System Grid Federation. Version 20200702. (2019). doi:https://doi.org/10.22033/ESGF/CMIP6.10115
61. Huang, W. THU CIESM model output prepared for CMIP6 CMIP historical. Earth System Grid Federation. Version 20200220. (2019). doi:https://doi.org/10.22033/ESGF/CMIP6.8843
62. Huang, W. THU CIESM model output prepared for CMIP6 ScenarioMIP ssp126. Earth System Grid Federation. Version 20200220. (2019). doi:https://doi.org/10.22033/ESGF/CMIP6.8857
63. Huang, W. THU CIESM model output prepared for CMIP6 ScenarioMIP ssp585. Earth System Grid Federation. Version 20200220. (2020). doi:https://doi.org/10.22033/ESGF/CMIP6.8863
64. Lovato, T., Peano, D. & Butenschön, M. CMCC CMCC-ESM2 model output prepared for CMIP6 CMIP historical. Earth System Grid Federation. Version 20210114. (2021). doi:https://doi.org/10.22033/ESGF/CMIP6.13195
65. Lovato, T., Peano, D. & Butenschön, M. CMCC CMCC-ESM2 model output prepared for CMIP6 ScenarioMIP ssp126. Earth System Grid Federation. Version 20210126. (2021). doi:https://doi.org/10.22033/ESGF/CMIP6.13250
66. Lovato, T., Peano, D. & Butenschön, M. CMCC CMCC-ESM2 model output prepared for CMIP6 ScenarioMIP ssp585. Earth System Grid Federation. Version 20210126. (2021). doi:https://doi.org/10.22033/ESGF/CMIP6.13259

67. Voldoire, A. CMIP6 simulations of the CNRM-CERFACS based on CNRM-CM6-1 model for CMIP experiment historical. Earth System Grid Federation. Version 20190125. (2018). doi:https://doi.org/10.22033/ESGF/CMIP6.4066
68. Voldoire, A. CNRM-CERFACS CNRM-CM6-1 model output prepared for CMIP6 ScenarioMIP ssp126. Earth System Grid Federation. Version 20190410. (2019). doi:https://doi.org/10.22033/ESGF/CMIP6.4184
69. Voldoire, A. CNRM-CERFACS CNRM-CM6-1 model output prepared for CMIP6 ScenarioMIP ssp585. Earth System Grid Federation. Version 20190410. (2019). doi:https://doi.org/10.22033/ESGF/CMIP6.4224
70. Seferian, R. CNRM-CERFACS CNRM-ESM2-1 model output prepared for CMIP6 CMIP historical. Earth System Grid Federation. Version 20190125. (2018). doi:https://doi.org/10.22033/ESGF/CMIP6.4068
71. Voldoire, A. CNRM-CERFACS CNRM-ESM2-1 model output prepared for CMIP6 ScenarioMIP ssp126. Earth System Grid Federation. Version 20190410. (2019). doi:https://doi.org/10.22033/ESGF/CMIP6.4186
72. Voldoire, A. CNRM-CERFACS CNRM-ESM2-1 model output prepared for CMIP6 ScenarioMIP ssp126. Earth System Grid Federation. Version 20190410. (2019). doi:https://doi.org/10.22033/ESGF/CMIP6.4186
73. Swart, N.C. et al. CCCma CanESM5 model output prepared for CMIP6 CMIP historical. Earth System Grid Federation. Version 20190429. (2019). doi:https://doi.org/10.22033/ESGF/CMIP6.3610
74. Swart, N.C. et al. CCCma CanESM5 model output prepared for CMIP6 ScenarioMIP ssp126. Earth System Grid Federation. Version 20190429. (2019). doi:https://doi.org/10.22033/ESGF/CMIP6.3683

75. Swart, N.C. et al. CCCma CanESM5 model output prepared for CMIP6 ScenarioMIP ssp585. Earth System Grid Federation. Version 20190429. (2019). doi:https://doi.org/10.22033/ESGF/CMIP6.3696
76. EC-Earth Consortium (EC-Earth). EC-Earth-Consortium EC-Earth3 model output prepared for CMIP6 CMIP historical. Earth System Grid Federation. Version 20200918. (2019). doi:https://doi.org/10.22033/ESGF/CMIP6.4700
77. EC-Earth Consortium (EC-Earth). EC-Earth-Consortium EC-Earth3 model output prepared for CMIP6 ScenarioMIP ssp126. Earth System Grid Federation. Version 20200918. (2019). doi:https://doi.org/10.22033/ESGF/CMIP6.4874
78. EC-Earth Consortium (EC-Earth). EC-Earth-Consortium EC-Earth3 model output prepared for CMIP6 ScenarioMIP ssp585. Earth System Grid Federation. Version 20200918. (2019). doi:https://doi.org/10.22033/ESGF/CMIP6.4912
79. Yu, Y. CAS FGOALS-f3-L model output prepared for CMIP6 CMIP historical. Earth System Grid Federation. Version 20191007. (2019). doi:https://doi.org/10.22033/ESGF/CMIP6.3355
80. Yu, Y. CAS FGOALS-f3-L model output prepared for CMIP6 ScenarioMIP ssp126. Earth System Grid Federation. Version 20191007. (2019). doi:https://doi.org/10.22033/ESGF/CMIP6.3464
81. Yu, Y. CAS FGOALS-f3-L model output prepared for CMIP6 ScenarioMIP ssp585. Earth System Grid Federation. Version 20191008. (2019). doi:https://doi.org/10.22033/ESGF/CMIP6.3502
82. Li, L. CAS FGOALS-g3 model output prepared for CMIP6 CMIP historical. Earth System Grid Federation. Version 20191012. (2019). doi:https://doi.org/10.22033/ESGF/CMIP6.3356

83. Li, L. CAS FGOALS-g3 model output prepared for CMIP6 ScenarioMIP ssp126. Earth System Grid Federation. Version 20200101. (2019). doi:https://doi.org/10.22033/ESGF/CMIP6.3465
84. Li, L. CAS FGOALS-g3 model output prepared for CMIP6 ScenarioMIP ssp585. Earth System Grid Federation. Version 20191230. (2019). doi:https://doi.org/10.22033/ESGF/CMIP6.3503
85. Song, Z. et al. FIO-QLNM FIO-ESM2.0 model output prepared for CMIP6 CMIP historical. Earth System Grid Federation. Version 20191218. (2019). doi:https://doi.org/10.22033/ESGF/CMIP6.9199
86. Song, Z. et al. FIO-QLNM FIO-ESM2.0 model output prepared for CMIP6 ScenarioMIP ssp126. Earth System Grid Federation. Version 20200110. (2019). doi:https://doi.org/10.22033/ESGF/CMIP6.9208
87. Song, Z. et al. FIO-QLNM FIO-ESM2.0 model output prepared for CMIP6 ScenarioMIP ssp585. Earth System Grid Federation. Version 20200114. (2019). doi:https://doi.org/10.22033/ESGF/CMIP6.9214
88. Ridley, J., Menary, M., Kuhlbrodt, T., Andrews, M. & Andrews, T. MOHC HadGEM3-GC31-LL model output prepared for CMIP6 CMIP historical. Earth System Grid Federation. Version 20190624. (2019). doi:https://doi.org/10.22033/ESGF/CMIP6.6109
89. Good, P. MOHC HadGEM3-GC31-LL model output prepared for CMIP6 ScenarioMIP ssp126. Earth System Grid Federation. Version 20200114. (2020). doi:https://doi.org/10.22033/ESGF/CMIP6.10849
90. Good, P. MOHC HadGEM3-GC31-LL model output prepared for CMIP6 ScenarioMIP ssp585. Earth System Grid Federation. Version 20200114. (2020). doi:https://doi.org/10.22033/ESGF/CMIP6.10901

91. Ridley, J., Menary, M., Kuhlbrodt, T., Andrews, M. & Andrews, T. MOHC HadGEM3-GC31-MM model output prepared for CMIP6 CMIP historical. Earth System Grid Federation. Version 20191216. (2019). doi:https://doi.org/10.22033/ESGF/CMIP6.6112
92. Jackson, L. MOHC HadGEM3-GC31-MM model output prepared for CMIP6 ScenarioMIP ssp126. Earth System Grid Federation. Version 20200515. (2020). doi:https://doi.org/10.22033/ESGF/CMIP6.10850
93. Jackson, L. MOHC HadGEM3-GC31-MM model output prepared for CMIP6 ScenarioMIP ssp585. Earth System Grid Federation. Version 20200515. (2020). doi:https://doi.org/10.22033/ESGF/CMIP6.10902
94. Boucher, O. et al. IPSL IPSL-CM6A-LR model output prepared for CMIP6 CMIP historical. Earth System Grid Federation. Version 20180803. (2018). doi:https://doi.org/10.22033/ESGF/CMIP6.5195
95. Boucher, O. et al. IPSL IPSL-CM6A-LR model output prepared for CMIP6 ScenarioMIP ssp126. Earth System Grid Federation. Version 20190305. (2019). doi:https://doi.org/10.22033/ESGF/CMIP6.5262
96. Boucher, O. et al. IPSL IPSL-CM6A-LR model output prepared for CMIP6 ScenarioMIP ssp585. Earth System Grid Federation. Version 20190903. (2019). doi:https://doi.org/10.22033/ESGF/CMIP6.5271
97. Tatebe, H. &; Watanabe, M. MIROC MIROC6 model output prepared for CMIP6 CMIP historical. Earth System Grid Federation. Version 20191121. (2018). doi:https://doi.org/10.22033/ESGF/CMIP6.5603
98. Shiogama, H., Abe, M. & Tatebe, H. MIROC MIROC6 model output prepared for CMIP6 ScenarioMIP ssp126. Earth System Grid Federation. Version 20190627. (2019). doi:https://doi.org/10.22033/ESGF/CMIP6.5743

99. Shiogama, H., Abe, M. & Tatebe, H. MIROC MIROC6 model output prepared for CMIP6 ScenarioMIP ssp585. Earth System Grid Federation. Version 20190627. (2019). doi:https://doi.org/10.22033/ESGF/CMIP6.5771

100. Jungclaus, J. et al. MPI-M MPI-ESM1.2-HR model output prepared for CMIP6 CMIP historical. Earth System Grid Federation. Version 20190710. (2019). doi:https://doi.org/10.22033/ESGF/CMIP6.6594

101. Schupfner, M. et al. DKRZ MPI-ESM1.2-HR model output prepared for CMIP6 ScenarioMIP ssp126. Earth System Grid Federation. Version 20190710. (2019). doi:https://doi.org/10.22033/ESGF/CMIP6.4397

102. Schupfner, M. et al. DKRZ MPI-ESM1.2-HR model output prepared for CMIP6 ScenarioMIP ssp585. Earth System Grid Federation. Version 20190710. (2019). doi:https://doi.org/10.22033/ESGF/CMIP6.4403

103. Wieners, K.-H. et al. MPI-M MPI-ESM1.2-LR model output prepared for CMIP6 CMIP historical. Earth System Grid Federation. Version 20190710. (2019). doi:https://doi.org/10.22033/ESGF/CMIP6.6595

104. Wieners, K.-H. et al. MPI-M MPI-ESM1.2-LR model output prepared for CMIP6 ScenarioMIP ssp126. Earth System Grid Federation. Version 20190710. (2019). doi:https://doi.org/10.22033/ESGF/CMIP6.6690

105. Wieners, K.-H. et al. MPI-M MPI-ESM1.2-LR model output prepared for CMIP6 ScenarioMIP ssp585. Earth System Grid Federation. Version 20190710. (2019). doi:https://doi.org/10.22033/ESGF/CMIP6.6705

106. Yukimoto, S. et al. MRI MRI-ESM2.0 model output prepared for CMIP6 CMIP historical. Earth System Grid Federation. Version 20210311. (2019). doi:https://doi.org/10.22033/ESGF/CMIP6.6842

107. Yukimoto, S. et al. MRI MRI-ESM2.0 model output prepared for CMIP6 ScenarioMIP ssp126. Earth System Grid Federation. Version 20210329. (2019). doi:https://doi.org/10.22033/ESGF/CMIP6.6909

108. Yukimoto, S. et al. MRI MRI-ESM2.0 model output prepared for CMIP6 ScenarioMIP ssp585. Earth System Grid Federation. Version 20210329. (2019). doi:https://doi.org/10.22033/ESGF/CMIP6.6929

109. Seland, Ø. et al. NCC NorESM2-LM model output prepared for CMIP6 CMIP historical. Earth System Grid Federation. Version 20190815. (2019). doi:https://doi.org/10.22033/ESGF/CMIP6.8036

110. Seland, Ø. et al. NCC NorESM2-LM model output prepared for CMIP6 ScenarioMIP ssp126. Earth System Grid Federation. Version 20191108. (2019). doi:https://doi.org/10.22033/ESGF/CMIP6.8248

111. Seland, Ø. et al. NCC NorESM2-LM model output prepared for CMIP6 ScenarioMIP ssp585. Earth System Grid Federation. Version 20191108. (2019). doi:https://doi.org/10.22033/ESGF/CMIP6.8319

112. Bentsen, M. et al. NCC NorESM2-MM model output prepared for CMIP6 CMIP historical. Earth System Grid Federation. Version 20191108. (2019). doi:https://doi.org/10.22033/ESGF/CMIP6.8040

113. Bentsen, M. et al. NCC NorESM2-MM model output prepared for CMIP6 ScenarioMIP ssp126. Earth System Grid Federation. Version 20191108. (2019). doi:https://doi.org/10.22033/ESGF/CMIP6.8250

114. Bentsen, M. et al. NCC NorESM2-MM model output prepared for CMIP6 ScenarioMIP ssp585. Earth System Grid Federation. Version 20191108. (2019). doi:https://doi.org/10.22033/ESGF/CMIP6.8321

115. Lee, W.-L. & Liang, H.-C. AS-RCEC TaiESM1.0 model output prepared for CMIP6 CMIP historical. Earth System Grid Federation. Version 20200630. (2020). doi:https://doi.org/10.22033/ESGF/CMIP6.9755

116. Lee, W.-L. & Liang, H.-C. AS-RCEC TaiESM1.0 model output prepared for CMIP6 ScenarioMIP ssp126. Earth System Grid Federation. Version 20201125. (2020). doi:https://doi.org/10.22033/ESGF/CMIP6.9806

117. Lee, W.-L. & Liang, H.-C. AS-RCEC TaiESM1.0 model output prepared for CMIP6 ScenarioMIP ssp585. Earth System Grid Federation. Version 20210416. (2020). doi:https://doi.org/10.22033/ESGF/CMIP6.9823

118. Tang, Y. et al. MOHC UKESM1.0-LL model output prepared for CMIP6 CMIP historical. Earth System Grid Federation. Version 20190502. (2019). doi:https://doi.org/10.22033/ESGF/CMIP6.6113

119. Good, P. et al. MOHC UKESM1.0-LL model output prepared for CMIP6 ScenarioMIP ssp126. Earth System Grid Federation. Version 20190507. (2019). doi:https://doi.org/10.22033/ESGF/CMIP6.6333

120. Good, P. et al. MOHC UKESM1.0-LL model output prepared for CMIP6 ScenarioMIP ssp585. Earth System Grid Federation. Version 20190507. (2019). doi:https://doi.org/10.22033/ESGF/CMIP6.6405

121. Swart, N. C. et al. CCCma CanESM5 model output prepared for CMIP6 ScenarioMIP rcp26-cmip5. Earth System Grid Federation. Version 20190429. (2019). doi:https://doi.org/10.22033/ESGF/CMIP6.12559

122. Swart, N. C., et al. CCCma CanESM5 model output prepared for CMIP6 ScenarioMIP rcp85-cmip5. Earth System Grid Federation. Version 20190429. (2019). doi:https://doi.org/10.22033/ESGF/CMIP6.12565
